# Does Litter Size Variation Affect Models of Terrestrial Carnivore Extinction Risk and Management?

**DOI:** 10.1371/journal.pone.0058060

**Published:** 2013-02-28

**Authors:** Eleanor S. Devenish-Nelson, Philip A. Stephens, Stephen Harris, Carl Soulsbury, Shane A. Richards

**Affiliations:** 1 School of Biological and Biomedical Sciences, Durham University, Durham, United Kingdom; 2 School of Biological Sciences, University of Bristol, Bristol, United Kingdom; 3 School of Life Sciences, University of Lincoln, Lincoln, United Kingdom; University of Toronto, Canada

## Abstract

**Background:**

Individual variation in both survival and reproduction has the potential to influence extinction risk. Especially for rare or threatened species, reliable population models should adequately incorporate demographic uncertainty. Here, we focus on an important form of demographic stochasticity: variation in litter sizes. We use terrestrial carnivores as an example taxon, as they are frequently threatened or of economic importance. Since data on intraspecific litter size variation are often sparse, it is unclear what probability distribution should be used to describe the pattern of litter size variation for multiparous carnivores.

**Methodology/Principal Findings:**

We used litter size data on 32 terrestrial carnivore species to test the fit of 12 probability distributions. The influence of these distributions on quasi-extinction probabilities and the probability of successful disease control was then examined for three canid species – the island fox *Urocyon littoralis*, the red fox *Vulpes vulpes*, and the African wild dog *Lycaon pictus*. Best fitting probability distributions differed among the carnivores examined. However, the discretised normal distribution provided the best fit for the majority of species, because variation among litter-sizes was often small. Importantly, however, the outcomes of demographic models were generally robust to the distribution used.

**Conclusion/Significance:**

These results provide reassurance for those using demographic modelling for the management of less studied carnivores in which litter size variation is estimated using data from species with similar reproductive attributes.

## Introduction

Demographic variation, resulting from extrinsic and intrinsic sources, fundamentally affects population dynamics and is particularly important when assessing extinction risk for threatened species [1], [2]. Predictions of population dynamics depend on the ability to attribute sources of stochasticity accurately in population models [3], [4]. Of particular importance is the distinction between demographic stochasticity and demographic heterogeneity. Demographic stochasticity is the random fate of an individual arising from a chance event drawn from a specified uniform vital rate, whereas demographic heterogeneity is the variation in the underlying parameter value arising from within-population variability in individual condition [5]. Both types of demographic variation make important contributions to a populations' total demographic variance [3]. Indeed, accounting for demographic stochasticity in fecundity can lead to increased predictions of extinction risk; for example, overall demographic variance is increased when this parameter is Poisson-distributed [5]. Here, we focus on stochasticity in demographic fates, which can easily be accounted for by drawing rates from appropriate probability distributions [6], [7].

Mean litter (or clutch) size has long been the focus of evolutionary and population biologists concerned with causes of interspecific variation [8]–[11], correlations with environmental gradients [9], [12]–[14] and optimality in this trait [15]–[18]. However, intra-population variation in litter size has been largely overlooked (but see [19]). Limited knowledge of the underlying measures of empirical litter size distributions, such as the degree of dispersion, hinders the accurate representation of the stochasticity of this parameter in population models. Demographic stochasticity in offspring number is most commonly modelled with Poisson or normal distributions [6], [20], [21], although there is little theoretical justification for these choices [19]. Furthermore, many demographic modelling programmes (e.g. RAMAS [7] and VORTEX [21]) have limited provision for specifying distributions. Unlike survival, which is a Bernoulli process [20], choosing a distribution to describe variation in litter sizes in multiparous species can be complex because the biology of reproduction differs substantially among species and is ultimately limited by physiological capacity. Standard probability distributions might lack the flexibility required to account for litter size variation in many species.

In population modelling, the influence of distribution choice has only been considered previously for demographic parameters other than litter size, with a focus on environmental stochasticity. Studies that modelled environmental stochasticity found that population growth rate (λ) estimates were underestimated as a result of inaccurately defined, symmetrical survival distributions [22] and large differences in λ estimates were found when drawing recruitment rates from different distributions [23]. Yet, the shape of the distribution may also be important for populations that are susceptible to fluctuations in vital rates as a result of demographic stochasticity, such as small populations. Failing to account for demographic stochasticity in litter size may lead to inaccurate predictions of extinction risk [19]. In this context, it is useful to establish whether failing to incorporate an appropriate theoretical distribution for litter size, to describe demographic stochasticity, could lead to erroneous estimates of model outputs.

Here, we examine the fit of specified candidate probability distributions to empirical data on terrestrial carnivore litter size frequencies. The Carnivora exhibit some of the most diverse life history traits of all mammalian orders, as reflected in their broad range of litter sizes [24]. While many carnivores are at increasing risk of extinction [25], others are predators of economic importance or important hosts of zoonotic and wildlife diseases such as rabies [26]. Although data collection is often challenging [27], both categories of carnivore are frequently the subject of population models (e.g. [28]–[30]). Given the importance of carnivore management and the sparseness of much of the data used to model carnivore demography, it is useful to establish whether the choice of distribution used to model demographic stochasticity in litter sizes affects the inferences drawn from models of carnivore population dynamics. To illustrate the applied importance of using appropriate distributions, three previously published population models are replicated to determine the consequences of mis-specifying litter size distributions for inferences regarding extinction probabilities or disease dynamics.

## Methods

### Probability distribution fitting

Litter size frequency data were collated for 32 terrestrial multiparous carnivore species, from 63 published studies of 73 wild populations, to reflect the diversity of life history within the order. Each species has a single annual breeding attempt. None of the studies included litters of zero; modelling litter size inherently assumes that an individual has bred. If studies presented data for multiple conspecific populations or for multiple methods of litter size determination, these were analysed as discrete datasets. For 15 species, data were obtained for between two and ten populations. For three species, data from multiple methods of litter size determination (e.g. placental scars and direct counts) were available. We thus also considered whether there was strong support for genuine underlying difference in litter size distributions between conspecific populations or between data determined by different methods (again, for a given population) (see Appendix S1 for details of the analyses).

Twelve probability distributions were selected based on a review of previous studies. Specifically, four discrete distributions were chosen: the Poisson distribution [6]; the generalised Poisson, which has a wide-ranging suitability for describing litter size frequencies [19]; the binomial distribution, previously fitted successfully to carnivore litter data [19]; and the negative binomial, widely used to describe ecological processes (e.g. [31]). For each discrete distribution, both a ‘right shifted’ and ‘zero-truncated’ form were fitted (Appendix S2), to exclude litter sizes of zero. For zero-truncation, the probability mass function was scaled by the exclusion of predicted zeros. Shifting involved moving the entire distribution one interval to the right. Three continuous probability distributions were chosen: the normal and lognormal distributions are both widely used [6], although log-transformation is not recommended for count data [32]; and the stretched beta (two and three parameter forms), as proposed by Morris and Doak [6]. Appendix S2 provides details of how these continuous distributions were converted into discrete forms.

Maximum-likelihood parameters, denoted 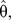 were estimated using the ‘optim’ function in R 2.14.0 (R Development Core Team 2011). Here, the multinomial log-likelihood defined by θ and given all the data is:
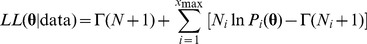
(1)where *N* is the total number of litters observed, *N_i_* is the number of litters observed of size *i*, *P_i_* is the predicted litter size probability determined by a given distribution (Appendix S2), *x_max_* is the maximum litter size, and Γ(x) is the complete gamma function. The fits for each probability distribution were compared using Akaike's Information Criterion (AIC); all distributions having a ΔAIC≤6 of the best fitting distribution (i.e. lowest AIC) were considered to have some support [33]. To check that our best-fitting models were consistent with the data, and because of the small sample sizes of the predicted frequencies, we performed goodness-of-fit tests using Fisher's Exact Test. Variance-mean ratios [34] were determined to measure the dispersion of the empirical and fitted distributions.

### Carnivore population models

Published stochastic population models for three management scenarios were used to illustrate the broader applied significance of this study. The Canidae were chosen because they provide the widest range of litter sizes within the Carnivora [24]. Models were chosen to depict a range of conservation and management scenarios that could be replicated from published data; the intention was to identify whether the choice of distribution used to represent litter sizes influences predicted model outcomes. By “outcomes”, we refer to a major emergent parameter from the models, on which further inference would be based (see below). The emergent parameter of interest varied because the three models were created for different applications. Using the parameters that were estimated by maximum likelihood as described above, 10,000 stochastic replicates of the models were simulated drawing litter sizes from each of the 12 probability distributions. This enabled calculation of 95% confidence intervals around mean outcome values. For each case study, disparities were determined between the outcome values of the 12 model versions. This allowed us to evaluate the effect on each model of employing different litter size distributions, in relation to the degree of empirical support for those distributions. See Appendix S3 for full descriptions of each case study model and Table 1 for the initial parameter values for the three models.

**Table 1:** Parameter values for the three population models.

*Initial parameter value*	*Model 1. Island fox*	*Model 2. Red fox*	*Model 3. African wild dog*
Quasi-extinction or disease density threshold	50	87% of initial population	One sex remains
Years	100	3	50
Time step	Annual	Monthly	Annual
Age at first reproduction	2	1	3
Sex ratio at birth	0.5	0.5	0.55
Dispersal age	1	1	-
Dispersal probability	0.01	*Female month 7–12*: 0.03, 0.030, 0.136, 0.045, 0.045, 0.030	-
		*Male month 7–12*: 0.68, 0.102, 0.182, 0.159, 0.102, 0.057	
Dispersal survival	0.8	-	-
Annual mortality rate pup	31.3±5.9	-	0.68±0.20
Annual mortality rate juvenile male	25.2±6.0	*Monthly*: 0.137, 0.045, 0.040, 0.048, 0.036, 0.035, 0.044, 0.044, 0.039, 0.062, 0.032, 0.035	0.20±0.03
Annual mortality rate juvenile female	16.8±4.7	*Monthly*: 0.129, 0.052, 0.067, 0.037, 0.042, 0.037, 0.044, 0.032, 0.039, 0.025, 0.034, 0.030	0.20±0.03
Annual mortality rate adult male	25.2±6.0	*Monthly*: 0.035, 0.039, 0.020, 0.028, 0.014, 0.039, 0.036, 0.046, 0.041, 0.121, 0.069, 0.029	0.15±0.03
Annual mortality rate adult female	16.8±4.7	*Monthly*: 0.041, 0.055, 0.035, 0.025, 0.023, 0.034, 0.044, 0.049, 0.035, 0.062, 0.041, 0.036	0.15±0.03
Probability of breeding	1	0.8	0.58 (dominant pairs only)
Density dependence in breeding (% breeding at carrying capacity)	*West subpopulation*: 58.38 East *subpopulation*: 55.03	-	-
Carry capacity, *K*	*West subpopulation*: 300	-	20
	*East subpopulation*: 1300		
Initial population size	*West subpopulation*: 90	1 male and 1 female per group,	20
	*East subpopulation*: 63	additional male or female added with probability of 0.80 and 0.58 additional individual 0.47 probability of being juvenile	
Disease Introduction	-	September	-
Incubation period	-	1 month	-
Probability of becoming rabid once exposed	-	0.42	-
Disease mortality	-	1	-
Control	-	40% control every 2 months, 3 months after disease introduction	-
Catastrophes	Frequency: 0.2	-	*Mild*: Frequency: 0.05
	Reduction in survival: 0.8		Survival reduction: 0.85
			Reproduction reduction: 0.5
			*Severe*: Frequency: 0.03
			Survival reduction: 0.5

First, we investigated the island fox *Urocyon littoralis,* which reached near extinction on Santa Catalina Island due to an outbreak of canine distemper virus [35]. We conducted a density-dependent population viability analysis (PVA) for two subpopulations, based on Kohlmann *et al.* [28]; the outcome of interest was the probability of quasi-extinction, defined in this model as the probability of the population declining to 50 individuals, due to a disease epidemic. Second, we investigated the red fox *Vulpes vulpes*, a locally abundant carnivore that is the focus of much attention due to its economic importance as a predator and role in the spread of rabies [36]. A density-dependent model simulating control after a rabies outbreak [29] was replicated to illustrate, as the outcome of interest, the probability of successful disease control. Finally, we investigated the African wild dog *Lycaon pictus*, which is restricted throughout much of its range and susceptible to several diseases, including rabies [37]. A density-dependent PVA [30] for small wild dog populations was reproduced to determine quasi-extinction probabilities (the outcome variable), defined here as the probability of only one sex remaining. Following Vial *et al*. [37], we also investigated the effects of including a component Allee effect (a positive relationship between population size and a measurable component of fitness [38]) with respect to recruitment. Here, rather than reducing pup mortality, individual litter size was assumed to be an increasing function of group size, *sensu* Vial *et al.* [37]. These three investigations illustrate canids with small, medium, and large mean litter sizes, respectively (Table S1). All modelling and analyses were conducted in R 2.14.0 (R Development Core Team 2011).

## Results

### Probability distribution fitting

**Figure 1:**
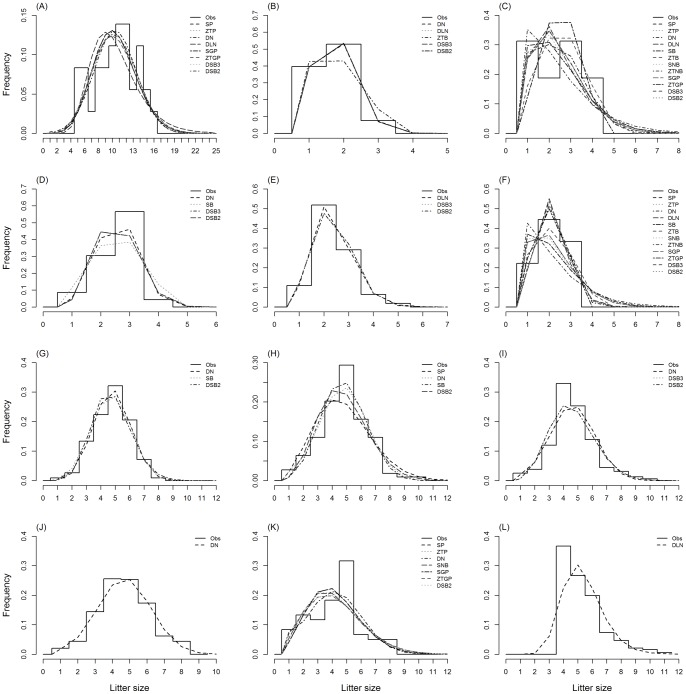
Observed litter size frequencies with fitted distributions with ΔAIC ≤6. The top two panels show for a range of sample sizes (of litters sampled), mean litter size, and carnivore families. The third panel from the top shows three populations of *Vulpes vulpes* with litter size determined by placental scars and the bottom panel illustrates three different methods for determining litter size of a Bristol population of *V. vulpes* (Harris, *unpublished data*). (A) *Lycaon pictus,* n = 36 [53]; (B) *Crocuta crocuta,* n = 108 [54]; (C) *Panthera tigris altaica*, n = 16 [55]; (D) *Ursus arctos,* n = 303 [56]; (E) *Meles meles*, n = 37 [57]; (F) *Lontra canadensis,* n = 9 [58]; (G) *V. vulpes*, n = 112 [59]; (H) *V. vulpes*, n = 506 [60]; (I) *V. vulpes*, London, n = 158 (Harris, *unpublished data*); (J) *V. vulpes*, placental scars, n = 340; (K) *V. vulpes*, embryos, n = 60; (L) *V. vulpes*, direct counts, n = 191. See Table S1 for details of datasets. Distribution abbreviations: observed frequencies (Obs); shifted Poisson (SP); ZT Poisson (ZTP); discretised normal (DN); discretised lognormal (DLN); discretised stretched beta –2 parameter form (DSB2); discretised stretched beta 3 parameter form (DSB3); shifted generalised Poisson (SGP); ZT generalised Poisson (ZTGP); shifted binomial (SB); ZT binomial (ZTB); shifted negative binomial (SNB); ZT negative binomial (ZTNB).

Variance-mean ratios of observed litter size frequencies (mean  = 0.41, SD ±0.40) indicated that empirical distributions tend to be underdispersed (Table S1). While the majority of datasets each represented one population (96%), most data were pooled over multiple years (97%) (Table S1). Best fitting distributions differed substantially between datasets (Tables 2 and S2), although all distributions with ΔAIC ≤6 provided fits consistent with the empirical data (Table S3). For 97% of all datasets, several of the 12 candidate distributions (mean  = 6.54, SD ±3.38) could not be discounted based on their AIC values (Table S2 and Fig. 1A–F for examples). The most widely applicable distribution was the discretised normal, with ΔAIC ≤6 for 95% of datasets; all other distributions were selected for between 22% and 87% of datasets. The “right shifted” method consistently performed better than zero-truncation for all distributions (Table S2), being on average 1.32 (SD ±0.16) times more likely to have a ΔAIC ≤6. While there was little support for intraspecific differences between red fox populations, distinct probability distributions best described litter size data determined by pre- and post-birth methods (Appendix S1 and Table S1).

**Table 2:** Model selection results for fitting probability distributions to carnivore litter size frequencies.

		*Distribution*											
*Family*	*Species*	*SP*	*ZTP*	*SB*	*ZTB*	*SNB*	*ZTNB*	*SGP*	*ZTGP*	*DN*	*DLN*	*DSB3*	*DSB2*
**Canidae**	*Vulpes velox*	1/1	-	1/1	-	-	-	1/1	-	**1/1**	1/1	1/1	1/1
	*Vulpes macrotis*	-	-	1/2	-	-	-	-	-	**2/2**	1/2	1/2	**2/2**
	*Vulpes vulpes*	**5/12**	2/12	4/12	4/12	2/12	-	4/12	2/12	**11/12**	**3/12**	6/12	7/12
	*Urocyon littoralis*	**1/2**	1/2	1/2	1/2	1/2	1/2	1/2	1/2	**1/2**	2/2	2/2	2/2
	*Urocyon cinereoargenteus*	1/2	1/2	1/2	1/2	1/2	1/2	1/2	1/2	2/2	**2/2**	2/2	**2/2**
	*Alopex lagopus*	-	-	1/3	-	1/3	-	**1/3**	1/3	2/3	**2/3**	3.3	3/3
	*Canis lupus*	**2/2**	2/2	1/2	1/2	1/2	2/2	2/2	2/2	**2/2**	1/2	2/2	2/2
	*Lycaon pictus*	**1/4**	1/4	-	-	1/4	1/4	3/4	**3/4**	**4/4**	2/4	4/4	3/4
	*Nyctereutes procyonoides*	1/1	1/1	1/1	1/1	-	-	1/1	-	**1/1**	1/1	1/1	1/1
**Hyaenidae**	*Crocuta crocuta*	-	-	1/3	1/3	-	-	-	-	**3/3**	3/3	3/3	**2/3**
**Procyonidae**	*Procyon lotor*	1/1	1/1	1/1	1/1	-	-	1/1	1/1	1/1	**1/1**	1/1	1/1
**Felidae**	*Acinonyx jubatus*	-	-	1/1	-	-	-	-	-	1/1	1/1	1/1	**1/1**
	*Felis concolor*	1/3	-	2/3	2/3	-	-	-	-	**3/3**	3/3	3/3	**3/3**
	*Felis iriomotensis*	**1/1**	1/1	-	-	1/1	1/1	1/1	1/1	1/1	1/1	1/1	1/1
	*Lynx pardinus*	-	-	1/1	-	-	-	-	-	1/1	**1/1**	1/1	1/1
	*Panthera tigris altaica*	**1/1**	1/1	-	1/1	1/1	1/1	1/1	1/1	1/1	1/1	1/1	1/1
	*Panthera onca*	**1/1**	1/1	-	1/1	1/1	1/1	1/1	1/1	1/1	1/1	1/1	-
	*Panthera leo*	2/6	-	**3/6**	1/6	-	-	2/6	-	**6/6**	**5/6**	6/6	**6/6**
	*Panthera pardus*	-	-	1/1	-	-	-	-	-	1/1	**1/1**	1/1	1/1
	*Leopardus pardalis*	**1/1**	1/1	1/1	1/1	1/1	1/1	1/1	-	1/1	1/1	1/1	1/1
**Ursidae**	*Ursus maritimus*	-	-	-	-	-	-	-	-	**4/4**	**4/4**	4/4	**4/4**
	*Ursus arctos*	-	-	**2/4**	-	-	-	-	-	**2/4**	3/4	31/4	**4/4**
	*Ursus americanus*	2/7	2/7	6/7	3/7	1/7	-	2/7	1/7	**7/7**	**5/7**	4/7	**5/7**
**Mustelidae**	*Lutra lutra*	**4/7**	2/7	**3/7**	4/7	3/7	1/7	4/7	1/7	**7/7**	7/7	7/7	**4/7**
	*Lontra canadensis*	**2/2**	2/2	**2/2**	2/2	2/2	1/2	2/2	2/2	2/2	2/2	2/2	2/2
	*Mustela erminea*	1/1	1/1	1/1	1/1	-	-	1/1	1/1	-	1/1	1/1	**1/1**
	*Mustela nigripes*	-	-	1/1	-	-	-	-	-	**1/1**	1/1	1/1	1/1
	*Martes pennanti*	-	-	-	-	-	-	-	-	1/1	**1/1**	1/1	-
	*Martes americana*	**1/1**	1/1	1/1	1/1	1/1	1/1	1/1	1/1	1/1	1/1	1/1	1/1
	*Spilogale putorius*	1/1	1/1	1/1	**1/1**	1/1	-	1/1	1/1	1/1	1/1	1/1	1/1
	*Gulo gulo*	-	-	1/1	1/1	-	-	-	-	**1/1**	1/1	1/1	-
	*Meles meles*	-	-	1/2	1/2	-	-	-	-	**1/2**	**2/2**	2/2	2/2

The number of datasets tested for each species (denominator, see Table S1 for details) and the number of datasets that were adequately fitted by a given distribution (numerator, see Table S2 for details). Bold indicates distributions that were most parsimonious for at least one dataset. SP: Shifted Poisson; ZTP: Zero-truncated Poisson; SB: Shifted binomial; ZTB: Zero-truncated binomial; SNB: Shifted negative binomial; ZTNB: Zero-truncated negative binomial; SGP: Shifted generalised Poisson; ZTGP: Zero-truncated generalised Poisson; DN: Discretised normal; DLN: Discretised lognormal; DSB3; Discretised stretched-beta (3 parameter form); DSB2; Discretised stretched-beta (2 parameter form).

### Carnivore population models

**Figure 2:**
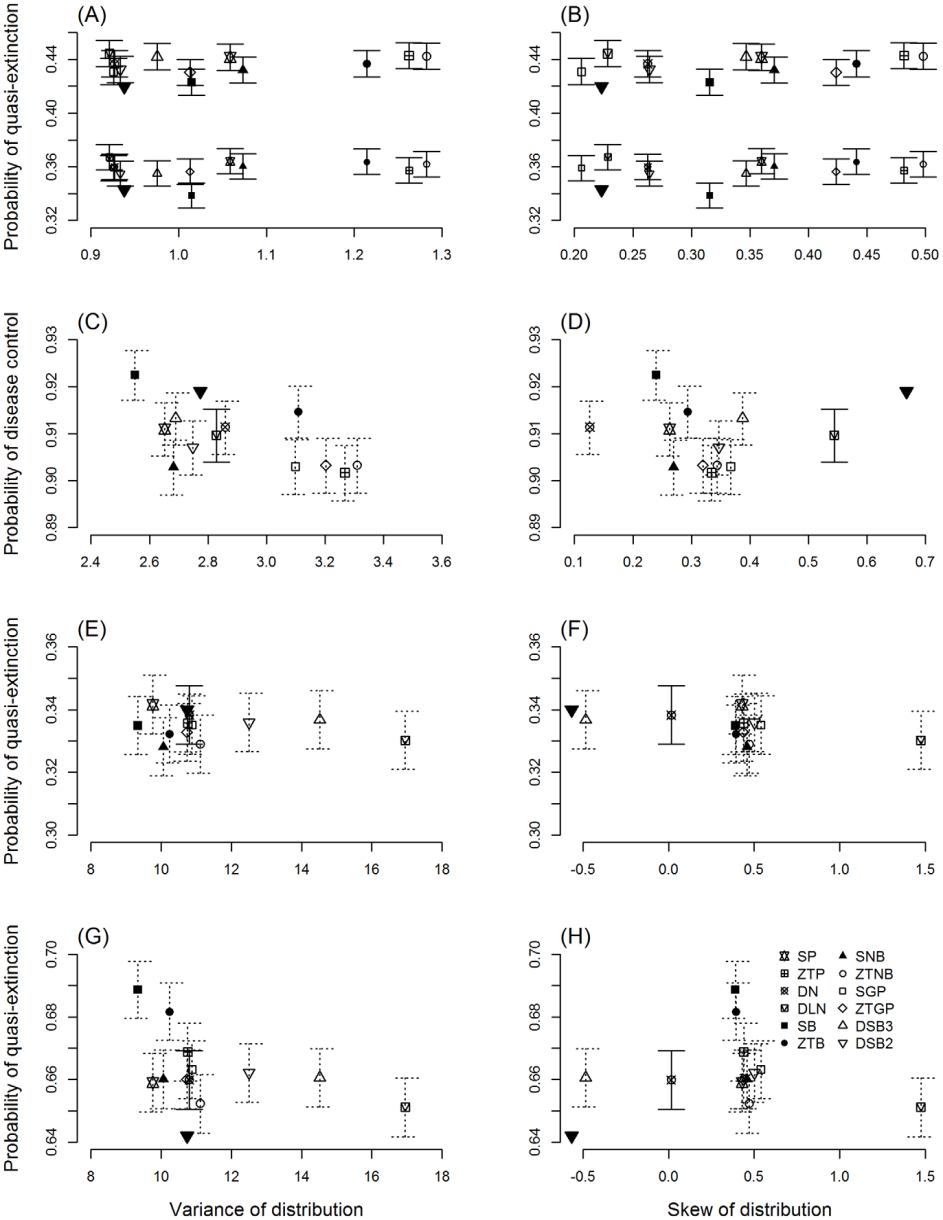
Model outcomes for 12 probability distributions against the variance (left panel) and skew (right panel) of distributions, showing quasi-extinction probabilities and probability of successful disease control, with 95% confidence intervals. (A, B) Island fox *Urocyon littoralis* PVA: west and east subpopulations; (C, D) red fox *Vulpes vulpes*; (E, F) African wild dog *Lycaon pictus* PVA without an Allee effect; (G, H) African wild dog PVA with an Allee effect included as a decrease in litter size as a function of group size. Solid error bars indicate distributions with ΔAIC ≤6. ▾ indicates the estimate from the previously published model, with the empirical litter size variance in the left panels and empirical litter size skew in the right panels (except G and H, for which there is no previous model estimate).

The demographic modelling showed that the distribution chosen to represent litter size uncertainty in the three canid models has limited impacts, regardless of the fit of the distributions. PVA models for island foxes showed that estimating extinction probability was largely unaffected by the choice of distribution, with less than 1% difference in quasi-extinction probabilities between models that used the best and worst fitting litter size distributions (Fig. 2A, B). Similarly, regardless of whether the litter size distributions used in the model provided a good fit to empirical litter size data, there was only a 2% difference in the probability of successful disease control in the rabies model for red foxes (Fig. 2C, D). Likewise, quasi-extinction probabilities for African wild dogs showed only a 1% difference among models that employed different litter size distributions (Fig. 2E, F). When litter size was reduced as a function of group size, to simulate an Allee effect, the influence of the distributions was slightly greater (Fig. 2G, H), with an increase of approximately 4% between quasi-extinction probabilities for the best and worst-fitting distributions. Even in this case, only models employing the worst-fitting distributions differed substantially in their predictions from those of models employing other distributions. The variation in the skew and variance of the fitted distributions (Fig. 2) may be attributed to process and sampling error in the data, as well as properties of the distributions such as the tendency to favour overdispersion, e.g. the negative binomial. However, for all parsimonious distributions, these measures were generally consistent with the empirical distributions for all models except island foxes (Fig. 2). In this latter case, the variation in agreement between distributions with ΔAIC ≤6 and the empirical properties (Fig. 2A, B) is probably due to the small sample size increasing the uncertainty of the observed parameter estimates, translating into the selection of multiple distributions. Despite the widely varying variance, the resultant model outcomes were in general unaltered by the choice of distribution. Coefficients of variation (CV) were small for all model outcomes (Table S4), with the greatest variation in the African wild dog model with an Allee effect; the best-fitting distribution (CV  = 0.712) was 1.07 times more variable than for the worst fitting model (CV  = 0.668).

## Discussion

Multiple distributions were shown to be consistent with the data for describing litter size frequencies for a range of carnivore species. However, the outcomes of demographic models appear robust to the choice of litter size distribution. These findings are discussed in light of the biological implications of litter size distribution choice and the applied importance of incorporating suitable probability distributions in demographic models.

### Model selection for describing litter size variation

Unlike many biological parameters, offspring number is often underdispersed [39], [40] and positively skewed [41], [42]. Litter size frequencies are best fitted by probability distributions able to describe the biological constraints on the upper limit of offspring production. While the Poisson distribution is most commonly used for fitting count data in general, it does not allow for underdispersion. In contrast, the generalised Poisson separates the variance from the mean [19], allowing greater flexibility, but at the cost of additional parameters. Of the continuous functions, the discretised normal distribution is the most flexible and is suitable for data characterised by low variance.

In a recent model of vertebrate reproductive success, the zero-truncated generalised Poisson was consistently the best-fitting of several parametric distributions fitted to litter size [19]. However, that study only included one carnivore population, (lion, *Panthera leo*), which was fitted solely by the zero-truncated-binomial. In our study, that distribution performed less well, perhaps because more competitive functions were considered (including shifted discrete distributions and discretised continuous distributions) that were not assessed in the earlier study [19]. The better fit of shifted forms over zero-truncation suggests that further work is needed to determine whether there is an underlying probabilistic mechanism in the distribution of litter size.

The lack of evidence for intraspecific variation in underlying litter size distributions (Appendix S1) could indicate that biological limitations on reproduction allow for little intraspecific variation in this trait. The known biases associated with litter size determination methods for red foxes [43], [44] probably explain the observed differences in litter size distributions (Appendix S1), although the results of the management scenarios analysed in this study (see next section) suggest that this finding is unlikely to be of consequence for future modelling efforts. Given the pooling of litter size datasets in this study over multiple years, due to insufficient data, the results must be interpreted with caution in light of potential temporal variation.

These analyses assumed that individuals had the same underlying expected reproductive capacity. However, demographic heterogeneity in offspring production is influenced by many factors, including female age, body condition or social status [45], [46], as well as maternal versus offspring trade-offs in reproductive success [47], [48]. The methods in these analyses could be incorporated into population models that address such intrinsic individual variation, as well as those modelling environmental stochasticity.

### Applied importance of litter size distributions

Despite interspecific variability in the consistency of distributions to describe litter size data, we have shown that model outcomes of applied management scenarios, e.g. extinction risk, may be robust to the distribution chosen to represent litter sizes. The lack of any apparent effect of litter size distribution choice in carnivore models might be because mammalian litter sizes are generally small due to physiological limitations. Underdispersion will promote sampling of offspring closer around the mean; therefore, sampling variation will only weakly impact model outcomes. There are indications that the distribution choice could be important in limited circumstances. In the case of African wild dog populations, the example presented here illustrates how modelling a component Allee effect in reproduction using an ill-fitting, underdispersed distribution can result in an overestimation of extinction risk (see Fig. 2E–H).

Further work is required to determine the potential influence of temporal variation in the underlying litter size distribution on predictions of extinction risk. This is particularly important given that temporal or environmental variability means that combining data over time will inflate estimates of litter size variation, leading to erroneous predictions of extinction risk. In spite of these concerns, the lack of available data meant that pooling data was necessary for our purposes; consequently, our results are indicative only of how mis-specified distributions could affect model predictions. As in [19], we stress that determining appropriate distributions is a step towards a more mechanistic understanding of litter size variability that could provide insight into a species' response to selective pressures or management actions.

That litter size distributions have limited effects on the outcomes of management models may also reflect the relative contributions of life history traits to population growth. For long-lived species such as carnivores [49], the elasticity of adult survival typically contributes more to population growth than fecundity. Indeed, variance in demographic parameters with low elasticities will have little effect on the variance of the population growth rate, due to the near linear relationship between population growth and vital rates [50]. Notably, for all three canid populations in the models presented here, the elasticity of survivorship is as high or higher than fecundity [28], [30], [51], which is consistent with the limited impact of litter size variation observed in the case studies.

Although this study focused on the Carnivora, our findings should apply to taxa with multiparous females, including other mammals, birds and lizards. While it is hard to determine the exact ecological and physiological mechanisms generating a litter size distribution, insight into the drivers of these empirical distributions could aid our understanding of the adaptation of reproductive strategies to extrinsic and intrinsic population pressures. Recent work demonstrating that female red foxes exhibit sex-biased investment in offspring as a function of body mass and population density suggests that altering litter size composition rather than litter size could be an alternative mechanism for increasing fitness [52]. Ultimately however, applied models for carnivores appear to be robust to choice of litter size distribution, which has positive implications for modelling species with limited data.

## Supporting Information

Table S1 **Summary of terrestrial carnivore litter size data from published studies.**
(DOC)

Table S2 **Model selection for 12 probability distributions fitted to carnivore litter size frequencies, with ΔAIC values.**
(DOC)

Table S3 **Results of the Fisher Exact test goodness-of-fit of probability distributions to empirical carnivore litter size frequencies.**
(DOC)

Table S4 **Coefficient of variation for model outcomes of quasi-extinction probabilities and probability of successful disease control, for 12 probability distributions.**
(DOC)

Appendix S1 **Testing for intraspecific variation in litter size distributions, using the red fox** ***Vulpes vulpes*** **as an example.**
(DOC)

Appendix S2 **Functional forms for the 12 probability distributions fitted to empirical litter size frequency data.**
(DOC)

Appendix S3 **Model descriptions for the three canid management scenarios used to illustrate the consequence of using different distributions to model litter size.**
(DOC)
