# Supplementary material for: Does Litter Size Variation Affect Models of Terrestrial Carnivore Extinction Risk and Management?
Source: PLoS One. 2013 Feb 28;8(2):e58060. doi: 10.1371/journal.pone.0058060 (PMC3585178; doi:10.1371/journal.pone.0058060)
Supplement: Table S1 — Summary of terrestrial carnivore litter size data from published studies. (DOC) [file pone.0058060.s001.doc]

Table S1. Summary of terrestrial carnivore litter size data from published studies.

The study duration in years and the number of populations* that the data refer to are indicated. The method of litter size determination refers to placental scars (ps), embryo counts (ec) or direct counts (dc). Sample size refers to the number of litters.

| ***Species[Reference]*** | ***Duration [Population]*** | ***Method*** | ***Sample size*** | ***Mean litter size*** | ***Variance*** | ***Variance/mean*** | ***Skewness*** |
| --- | --- | --- | --- | --- | --- | --- | --- |
| *Vulpes velox* ^[1]^ | 2 [1] | dc | 9 | 4.78 | 0.840 | 0.176 | -0.126 |
| *Vulpes macrotis* ^[2]^ | 15 [1] | dc | 101 | 3.75 | 1.632 | 0.435 | -0.078 |
| *Vulpes macrotis* ^[3]^ | 4 [1] | dc | 50 | 4.55 | 0.248 | 0.055 | -0.024 |
| *Vulpes vulpes* ^[4]^ | 4 [1] | ps | 112 | 4.77 | 1.660 | 0.348 | -0.072 |
| *Vulpes vulpes* ^[5]^ | 6 [1] | ec | 114 | 4.46 | 2.774 | 0.623 | 0.285 |
| *Vulpes vulpes* ^[6]^ | 14 [1] | dc | 106 | 4.85 | 3.713 | 0.766 | 0.220 |
| *Vulpes vulpes* ^[7]^ | 6 [1] | ec | 114 | 5.05 | 2.576 | 0.510 | -0.125 |
| *Vulpes vulpes* ^[8]^ | 3 [1] | ps | 113 | 4.40 | 4.877 | 1.109 | 0.144 |
| *Vulpes vulpes* ^[8]^ | 4 [1] | ps | 58 | 4.29 | 2.104 | 0.490 | 0.106 |
| *Vulpes vulpes* ^[8]^ | 4 [1] | ps | 109 | 4.79 | 2.754 | 0.575 | 0.059 |
| *Vulpes vulpes* ^[9]^ | 6 [1] | ps | 158 | 4.62 | 2.375 | 0.514 | 0.243 |
| *Vulpes vulpes* ^[10]^ | 6 [1] | ec | 42 | 5.05 | 1.807 | 0.358 | 0.015 |
| *Vulpes vulpes* ^[11]^ | 13 [1] | ps | 340 | 4.69 | 2.351 | 0.502 | 0.066 |
| *Vulpes vulpes* ^[12]^ | 13 [1] | ec | 60 | 4.17 | 3.206 | 0.769 | 0.046 |
| *Vulpes vulpes* ^[13]^ | 17 [1] | dc | 191 | 5.35 | 2.332 | 2.332 | 0.667 |
| *Urocyon littoralis* ^[14]^ | 2 [1] | dc | 20 | 2.50 | 0.550 | 0.220 | -0.089 |
| *Urocyon littoralis* ^[15]^ | 5 [1] | ec | 34 | 2.06 | 0.938 | 0.455 | 0.223 |
| *Urocyon cinereoargenteus* ^[16]^ | 2 [1] | ec | 7 | 3.14 | 0.980 | 0.312 | 0.185 |
| *Urocyon cinereoargenteus* ^[17]^ | 5 [1] | ps | 98 | 4.56 | 1.185 | 0.260 | 0.082 |
| *Alopex lagopus* ^[18]^ | 19 [1] | dc | 167 | 6.31 | 11.003 | 1.745 | 1.233 |
| *Alopex lagopus* ^[19]^ | 3 [1] | dc | 17 | 6.41 | 1.772 | 0.276 | 0.219 |
| *Canis lupus* ^[20]^ | 4 [1] | ps | 12 | 5.42 | 3.076 | 0.568 | 0.552 |
| *Canis lupus* ^[21]^ | 12 [1] | dc | 26 | 5.27 | 4.658 | 0.884 | 0.080 |
| *Lycaon pictus* ^[22]^ | Multiple [Multiple] | dc | 246 | 10.69 | 11.156 | 1.044 | -0.539 |
| *Lycaon pictus* ^[23]^ | 15 [1] | dc | 39 | 10.31 | 26.162 | 2.538 | -0.565 |
| *Lycaon pictus* ^[23]^ | 15 [1] | dc | 36 | 10.39 | 9.293 | 0.895 | 1.654 |
| *Lycaon pictus* ^[23]^ | 6 [1] | dc | 25 | 8.88 | 16.746 | 1.886 | -0.377 |
| *Nyctereutes procyonoides* ^[24]^ | 4 [1] | ps | 15 | 8.13 | 2.916 | 0.358 | 0.077 |
| *Procyon lotor* ^[25]^ | 3 [1] | dc | 15 | 8.13 | 2.916 | 0.358 | -0.864 |
| *Crocuta crocuta* ^[26]^ | 3 [1] | dc | 53 | 1.68 | 0.369 | 0.220 | 0.043 |
| *Crocuta crocuta* ^[26]^ | 3 [1] | dc | 55 | 1.56 | 0.246 | 0.157 | -0.027 |
| *Crocuta crocuta* ^[27]^ | 8 [1] | dc | 106 | 1.44 | 0.266 | 0.184 | 0.047 |
| *Acinonyx jubatus* ^[28]^ | 3 [1] | dc | 21 | 3.76 | 0.753 | 0.200 | 0.594 |
| *Felis concolor* ^[29]^ | 9 [1] | dc | 26 | 2.38 | 0.621 | 0.261 | 0.013 |
| *Felis concolor* ^[30]^ | 9[ 1] | dc | 27 | 2.22 | 0.469 | 0.211 | -0.079 |
| *Felis concolor* ^[31]^ | 18 [Multiple] | dc | 258 | 2.87 | 0.825 | 0.287 | -0.058 |
| *Felis iriomotensis* ^[32]^ | 13 [1] | dc | 41 | 1.10 | 0.088 | 0.080 | 0.058 |
| *Lynx pardinus* ^[33]^ | 9 [1] | dc | 15 | 3.13 | 0.516 | 0.165 | 0.179 |
| *Panthera tigris altaica* ^[34]^ | 8 [1] | dc | 16 | 2.38 | 1.234 | 0.520 | 0.018 |
| *Panthera onca* ^[35]^ | 2 [1] | dc | 23 | 1.61 | 0.499 | 0.310 | 0.136 |
| *Panthera leo* ^[36]^ | 2 [1] | dc | 34 | 2.32 | 0.807 | 0.347 | 0.203 |
| *Panthera leo* ^[37]^ | 4 [1] | dc | 28 | 2.68 | 0.504 | 0.188 | -0.010 |
| *Panthera leo* ^[37]^ | 4 [1] | dc | 38 | 2.82 | 0.677 | 0.240 | 0.017 |
| *Panthera leo* ^[38]^ | 24 [1] | dc | 110 | 2.54 | 1.049 | 0.413 | 0.044 |
| *Panthera leo* ^[38]^ | 24 [1] | dc | 200 | 2.46 | 1.028 | 0.418 | 0.151 |
| *Panthera leo* ^[38]^ | 24 [1] | dc | 159 | 2.48 | 1.130 | 0.455 | 0.144 |
| *Panthera pardus* ^[39]^ | 5 [1] | dc | 11 | 1.73 | 0.198 | 0.115 | 0.130 |
| *Leopardus pardalis* ^[40]^ | 13 [1] | dc | 13 | 1.23 | 0.178 | 0.144 | 0.141 |
| *Ursus maritimus* ^[41]^ | 26 [1] | dc | 261 | 1.89 | 0.336 | 0.178 | -0.101 |
| *Ursus maritimus* ^[42]^ | 3 [1] | dc | 61 | 1.74 | 0.193 | 0.111 | 0.001 |
| *Ursus maritimus* ^[42]^ | 2 [1] | dc | 44 | 1.86 | 0.163 | 0.088 | -0.081 |
| *Ursus maritimus* ^[42]^ | 1 [1] | dc | 15 | 2.27 | 0.329 | 0.145 | 0.299 |
| *Ursus arctos* ^[43]^ | 17 [1] | dc | 46 | 2.56 | 0.507 | 0.197 | -0.095 |
| *Ursus arctos* ^[43]^ | 17 [1] | dc | 51 | 2.06 | 0.487 | 0.236 | -0.108 |
| *Ursus arctos* ^[43]^ | 16 [1] | dc | 91 | 2.09 | 0.476 | 0.228 | -0.014 |
| *Ursus arctos* ^[44]^ | 43 [1] | dc | 56 | 2.39 | 0.524 | 0.219 | 0.014 |
| *Ursus americanus* ^[45]^ | 4 [1] | dc | 15 | 2.53 | 0.516 | 0.204 | 0.073 |
| *Ursus americanus* ^[46]^ | 16 [1] | dc | 86 | 2.35 | 0.599 | 0.255 | 0.184 |
| *Ursus americanus* ^[47]^ | 4 [1] | dc | 12 | 2.75 | 0.688 | 0.250 | -0.047 |
| *Ursus americanus* ^[48]^ | 4 [1] | dc | 23 | 1.65 | 0.401 | 0.243 | -0.111 |
| *Ursus americanus* ^[49]^ | 4 [1] | dc | 50 | 2.41 | 0.538 | 0.223 | 0.084 |
| *Ursus americanus* ^[50]^ | 12 [1] | dc | 105 | 2.49 | 0.593 | 0.238 | 0.065 |
| *Ursus americanus* ^[51]^ | 3 [1] | dc | 35 | 2.74 | 0.477 | 0.174 | 0.009 |
| *Lutra lutra* ^[52]^ | Multiple [Multiple] | dc | 160 | 2.45 | 0.346 | 0.847 | 0.124 |
| *Lutra lutra* ^[53]^ | 11 [Multiple] | ec | 17 | 2.06 | 0.526 | 0.255 | -0.021 |
| *Lutra lutra* ^[54]^ | 5 [1] | dc | 28 | 1.64 | 0.515 | 0.314 | 0.260 |
| *Lutra lutra* ^[55]^ | 50 [7] | ps | 30 | 2.27 | 0.662 | 0.292 | 0.057 |
| *Lutra lutra* ^[55]^ | 50 [7] | dc | 121 | 2.39 | 0.833 | 0.349 | 0.082 |
| *Lutra lutra* ^[55]^ | 50 [7] | ec | 46 | 2.02 | 0.673 | 0.333 | 0.166 |
| *Lontra canadensis* ^[56]^ | 10 [Multiple] | ec | 9 | 2.11 | 0.543 | 0.257 | 0.148 |
| *Mustela erminea* ^[57]^ | 4 [Multiple] | ec | 12 | 8.58 | 2.910 | 0.339 | -0.030 |
| *Mustela nigripes* ^[58]^ | 4 [1] | dc | 68 | 3.29 | 0.796 | 0.242 | 0.074 |
| *Martes pennanti* ^[59]^ | 6 [1] | ec | 9 | 3.33 | 0.222 | 0.067 | 0.053 |
| *Martes americana* ^[60]^ | 5 [1] | dc | 10 | 1.40 | 0.240 | 0.171 | -0.210 |
| *Spilogale putorius* ^[61]^ | 1 [1] | dc | 12 | 3.58 | 1.576 | 0.440 | -0.024 |
| *Gulo gulo* ^[62]^ | 19 [Multiple] | dc | 28 | 2.46 | 0.606 | 0.246 | -0.025 |
| *Meles meles* ^[63]^ | Multiple [Multiple] | ps | 37 | 2.95 | 0.869 | 0.295 | 0.142 |
| *Meles meles* ^[63]^ | Multiple [Multiple] | obs | 23 | 2.36 | 0.686 | 0.290 | -0.126 |
| **Mean ± SD** |  |  | 67.89 ± 67.953 | 3.52 ± 2.131 | 1.99 ± 3.872 | 0.40 ± 0.394 | 0.074 ± 0.310 |

*A population is defined by that described in a given study.

**References cited only in Table S1**

1. Kilgore DLJ (1969) An ecological study of the swift fox (*Vulpes velox*) in the Oklahoma panhandle. Am Midl Nat 81: 512-534.

2. Cypher BL, Warrick GD, Otten MRM, O'Farrell TP, Berry WH, et al. (2000) Population dynamics of San Joaquin kit foxes at the naval petroleum reserves in California. Wildl Monogr 145: 1-43.

3. Egoscue HJ (1962) Ecology and life history of the kit fox in Tooele county, Utah. Ecology 43: 481-497.

4. Vos AC (1994) Reproductive performance of the red fox, *Vulpes vulpes*, in Garmisch-Partenkirchen, Germany, 1987-1992. Z Säugetier 59: 326-331.

5. Lloyd HG (1980) The red fox. London: Batsford.

6. Stubbe M (1980) Population ecology of the red fox (*Vulpes vulpes* L., 1758) in the G.D.R. Biogeographica 18: 71-96.

7. Allen SH (1984) Some aspects of reproductive performance in female red fox in North Dakota. J Mammal 65: 246-255.

8. Englund J (1970) Some aspects of reproduction and mortality rates in Swedish foxes (*Vulpes vulpes*), 1961-63 and 1966-69. Viltrevy 8(1): 1-82.

9. Harris S (*unpublished data*) London population (placental scars).

10. Harris S (*unpublished data*) London population (embryo counts).

11. Harris S (*unpublished data*) Bristol population (placental scars).

12. Harris S (*unpublished data*) Bristol population (embryo counts).

13. Harris S (*unpublished data*) direct counts.

14. Clifford DL, Mazet JAK, Dubovi EJ, Garcelon DK, Coonan TJ, et al. (2006) Pathogen exposure in endangered island fox (*Urocyon littoralis*) populations: implications for conservation management. Biol Conserv 131: 230-243.

15. Coonan T (*unpublished data*).

16. Weston Glenn JL, Civitello DJ, Lance SL (2009) Multiple paternity and kinship in the gray fox (*Urocyon cinereoargenteus*). Z Säugetier 74: 394–402.

17. Wood JE (1958) Age structure and productivity of a gray fox population. J Mammal 39: 74-86.

18. Angerbjörn A, Tannerfeldt M, Bjärvall A, Ericson M, From J, et al. (1995) Dynamics of the arctic fox population in Sweden. Ann Zool Fenn 32: 55-68.

19. Barabash-Nikiforov I (1938) Mammals of the Commander Islands and the surrounding sea. J Mammal 19: 423-429.

20. McNay ME, Stephenson TR, Dale BW (2006) Diagnosing pregnancy, in utero litter size, and fetal growth with ultrasound in wild, free-ranging wolves. J Mammal 87: 85–92.

21. Pletscher DH, Ream RR, Boyd DK, Fairchild MW, Kunkel KE (1997) Population dynamics of a recolonizing wolf population. J Wildl Manag 61: 459-465.

22. Ginsberg JR, Woodroffe R (1997) Extinction risks faced by remaining wild dog populations. In: Woodroffe R, Ginsberg J, Macdonald D, editors. The African wild dog: status survey and conservation action plan. Gland: IUCN. pp. 75-87.

23. Creel S, Mills MGL, McNutt JW (2004) Demography and population dynamics of African wild dogs in three critical populations. In: Macdonald DW, Sillero-Zubiri C, editors. The biology and conservation of wild canids. Oxford: Oxford University Press. pp. 337-350.

24. Kowalczyk R, Zalewski A, Jedrzejewska B, Ansorge H, Bunevich AN (2009) Reproduction and mortality of invasive raccoon dogs (*Nyctereutes procyonoides*) in the Bialowieza primeval forest (eastern Poland). Ann Zool Fenn 46: 291-301.

25. Stuewer FW (1943) Reproduction of raccoons in Michigan. J Wildl Manag 7: 60-73.

26. Watts HE, Holekamp KE (2008) Interspecific competition influences reproduction in spotted hyenas. J Zool 276: 402–410.

27. Holekamp KE, Smale L, Szykman M (1996) Rank and reproduction in the female spotted hyaena. J Repro Fert 108: 229-237.

28. Laurenson ML (1995) Cub growth and maternal care in cheetahs. Behav Ecol 6: 405-409.

29. Lindzey FG, Van Sickle WD, Ackerman BB, Barnhurst D, Hemker TP, et al. (1994) Cougar population dynamics in southern Utah. J Wildl Manag 58: 619-624.

30. Ross PI, Jalkotzy MG (1992) Characteristics of a hunted population of cougars in southwestern Alberta. J Wildl Manag 56: 417-426.

31. Robinette WL, Gashwiler JS, Morris OW (1961) Notes on cougar productivity and life history. J Mammal 42: 204-217

32. Okamura M, Doi T, Sakaguchi N, Izawa M (2000) Annual reproductive cycle of the Iriomote cat *Felis iriomotensis*. Mamm Study 25: 75-85.

33. Palomares F, Revilla E, Calzada J, Fernández N, Delibes M (2005) Reproduction and pre-dispersal survival of Iberian lynx in a subpopulation of the Doñana National Park. Biol Conserv 122: 53–59.

34. Kerley LL, Goodrich JM, Miquelle DG, Smirnov EN, Quigley HB, et al. (2003) Reproductive parameters of wild female Amur (siberian) tigers (*Panthera tigris altaica*). J Mammal 84: 288-298.

35. Rabinowitz A (1986) Jaguar. New York: Arbor House. 368 p.

36. Schaller GB (1972) The Serengeti Lion. Chicago: University of Chicago Press. 480 p.

37. Hanby JP, Bygott JD, Packer C (1995) Ecology, demography and behaviour of lions in two contrasting habitats: Ngorongoro crator and the Serengeti plains. In: Sinclair ARE, Arcese P, editors. Serengeti II: dynamics, management, and conservation of an ecosystem. Chicago: University of Chicago Press. pp. 315-331.

38. Packer C, Pusey AE (1995) The Lack clutch in a communal breeder: lion litter size is a mixed evolutionarily stable strategy. Am Nat 145: 833-841.

39. Owen C, Niemann S, Slotow R (2010) Copulatory parameters and reproductive success of wild leopards in South Africa. J Mammal 91: 1178-1187.

40. Laack LL, Tewes ME, Haines AM, Rappole JH (2005) Reproductive life history of ocelots *Leopardus pardalis* in southern Texas. Acta Theriol 50: 505–514.

41. Derocher AE, Stirling I (1998) Maternal investment and factors affecting offspring size in polar bears (*Ursus maritimus*). J Zool 245: 253-260.

42. Larsen T (1985) Polar bear denning and cub production in Svalbard, Norway. J Wildl Manag 49: 320-326.

43. Miller SD, Sellers RA, Keay JA (2003) Effects of hunting on brown bear cub survival and litter size in Alaska. Ursus 14: 130-152.

44. Frković A, Huber D, Kusak J (2001) Brown bear litter sizes in Croatia. Ursus 12: 103-105.

45. Samson C, Huot J (1995) Reproductive biology of female black bears in relation to body mass in early winter. J Mammal 76: 68-77.

46. McDonald JEJ, Fuller TK (2001) Prediction of litter size in American black bears. Ursus 12: 93-102.

47. Doan-Crider DL, Hellgren EC (1996) Population characteristics and winter ecology of black bears in Coahuila, Mexico. J Wildl Manag 60: 398-407.

48. Beecham JJ (1980) Some population characteristics of two black bear populations in Idaho. Bears: their biology and management 4: 201-204.

49. Eiler JH, Wathen WG, Pelton MR (1989) Reproduction in black bears in the southern Appalachian mountains. J Wildl Manag 53: 353-360.

50. Noyce KV, Garshelis DL (1994) Body size and blood characteristics as indicators of condition and reproductive performance in black bears. Bears: their biology and management 9: 481-496.

51. Rogers L (1976) Effects of mast and berry crop failures on survival, growth, and reproductive success of black bears. Trans N Am Wildl Nat Resour Conf 41: 431-438.

52. Mason CF, Macdonald SM (1986) Otters: ecology and conservation. Cambridge: Cambridge University Press. 245 p.

53. Heggberget TM (1988) Reproduction in the female European otter in central and northern Norway. J Mammal 69: 164-167.

54. Kruuk HH, Conroy JWH, Moorhouse A (1991) Recruitment to a population of otters (*Lutra lutra*) in Shetland, in relation to fish abundance. J Appl Ecol 28: 95-101.

55. Hauer S, Ansorge H, Zinke O (2002) Reproductive performance of otters *Lutra lutra* (Linnaeus, 1758) in Eastern Germany: low reproduction in a long-term strategy. Biol J Linn Soc 77: 329-340.

56. Hamilton WJJ, Eadie WR (1964) Reproduction in the otter, *Lutra canadensis*. J Mammal 45: 242-252.

57. Deanesly R (1935) The reproductive processes of certain mammals. Part IX. Growth and reproduction in the stoat (*Mustela erminea*). Philos Trans R Soc Lond B Biol Sci 225: 459-492.

58. Forrest SC, Biggins DE, RIchardson L, Clark TW, Campbell TM, et al. (1988) Population attributes for the black-footed ferret (*Mustela nigripes*) at Meeteetse, Wyoming, 1981-1985. J Mammal 69: 261-273.

59. Wright PL, Coulter MW (1967) Reproduction and growth in Maine fishers. J Wildl Manag 31: 70-87.

60. Henry SE, O'Doherty EC, Ruggiero LF, van Sickle WD (1997) Maternal den attendance patterns of female American martens. In: Proulx G, Bryant HN, Woodard PM, editors. Martes: taxonomy, ecology, techniques, and management. Edmonton: University of Alberta Press. pp. 78-85.

61. Mead RA (1968) Reproduction in western forms of the spotted skunk (genus *Spilogale*). J Mammal 49: 373-390.

62. Pulliainen E (1968) Breeding biology of the wolverine (*Gulo gulo* L.) in Finland. Ann Zool Fenn 5: 338-344.

63. Neal EG, Cheeseman C (1996) Badgers. London: Poyser.
