# Supplementary material for: Does Litter Size Variation Affect Models of Terrestrial Carnivore Extinction Risk and Management?
Source: PLoS One. 2013 Feb 28;8(2):e58060. doi: 10.1371/journal.pone.0058060 (PMC3585178; doi:10.1371/journal.pone.0058060)
Supplement: Table S2 — Model selection for 12 probability distributions fitted to carnivore litter size frequencies, with ΔAIC values. (DOC) [file pone.0058060.s002.doc]

Table S2. Model selection for 12 probability distributions fitted to carnivore litter size frequencies, showing ΔAIC values. Bold indicates the distributions for which ΔAIC ≤ 6. References refer to those in Table S1. Distribution abbreviations: SP: Shifted Poisson; ZTP: Zero-truncated Poisson; SB: Shifted binomial; ZTB: Zero-truncated binomial; SNB: Shifted negative binomial; ZTNB: Zero-truncated negative binomial; SGP: Shifted generalised Poisson; ZTGP: Zero-truncated generalised Poisson; DN: Discretised normal; DLN: Discretised lognormal; DSB3; Discretised stretched-beta (3 parameter form); DSB2; Discretised stretched-beta (2 parameter form).

|  | ***Distribution*** | | | | | | | | | | | |
| --- | --- | --- | --- | --- | --- | --- | --- | --- | --- | --- | --- | --- |
| ***Species^(reference)^*** | ***SP*** | ***ZTP*** | ***SB*** | ***ZTB*** | ***SNB*** | ***ZTNB*** | ***SGP*** | ***ZTGP*** | ***DN*** | ***DLN*** | ***DSB3*** | ***DSB2*** |
| *Vulpes velox^1^* | **4.79** | 6.29 | **1.89** | **2.03** | 6.40 | 7.81 | 7.00 | 8.57 | **0.00** | **0.71** | **1.83** | **0.12** |
| *Vulpes macrotis^2^* | 13.56 | 23.17 | **1.87** | **0.00** | 12.20 | 21.33 | 16.51 | 26.63 | **0.10** | 17.01 | **0.09** | 7.64 |
| *Vulpes macrotis^3^* | 18.17 | 20.42 | 12.41 | 12.79 | 19.62 | 21.83 | 20.53 | 22.86 | **0.00** | **0.00** | **2.00** | **0.00** |
| *Vulpes vulpes^4^* | 29.70 | 43.24 | **0.46** | **0.22** | 28.35 | 41.20 | 33.17 | 47.25 | **0.00** | 15.00 | **2.79** | 6.71 |
| *Vulpes vulpes^5^* | **0.51** | 6.78 | **0.00** | **0.10** | **1.39** | 6.17 | **2.91** | 9.61 | **0.68** | 8.45 | **5.73** | **4.07** |
| *Vulpes vulpes^6^* | **0.00** | **2.63** | **1.93** | **1.95** | **1.95** | **3.50** | **2.09** | **5.14** | **4.46** | 7.71 | **2.16** | **2.00** |
| *Vulpes vulpes^7^* | 12.86 | 20.61 | **3.39** | **1.12** | 12.73 | 18.70 | 15.91 | 24.25 | **0.00** | 23.27 | **1.17** | 10.53 |
| *Vulpes vulpes^8^* | 22.19 | 9.02 | 14.21 | 9.14 | 25.19 | 11.99 | 23.39 | 10.63 | **0.00** | 25.08 | **5.57** | 12.61 |
| *Vulpes vulpes^8^* | **3.69** | 8.80 | **0.43** | **0.25** | **4.52** | 8.97 | 6.12 | 11.50 | **0.00** | **5.45** | **3.48** | **2.63** |
| *Vulpes vulpes^8^* | **5.74** | 11.97 | **2.41** | **1.05** | 6.13 | 11.51 | 8.31 | 14.97 | **0.00** | 16.85 | 7.98 | 8.85 |
| *Vulpes vulpes^9^* | 9.77 | 23.18 | **0.00** | **0.96** | 8.65 | 17.44 | 12.77 | 26.78 | **0.21** | 9.57 | 6.85 | **5.15** |
| *Vulpes vulpes^10^* | 9.11 | 14.21 | **0.00** | **0.17** | 9.81 | 14.96 | 11.70 | 17.01 | **0.26** | **2.43** | **1.46** | **0.39** |
| *Vulpes vulpes^11^* | 31.15 | 59.49 | **2.21** | **1.13** | 25.75 | 43.60 | 35.43 | 65.10 | **0.00** | 35.10 | 12.17 | 17.23 |
| *Vulpes vulpes^12^* | **2.24** | **1.38** | **4.24** | **2.19** | **4.38** | **2.91** | **4.24** | **3.64** | **0.00** | 11.84 | **3.37** | **5.59** |
| *Vulpes vulpes^13^* | 51.26 | 69.99 | 46.43 | 71.87 | 57.63 | 72.36 | 52.29 | 72.19 | 157.74 | **0.00** | 8.81 | 16.22 |
| *Urocyon littoralis^14^* | 6.43 | 9.66 | **2.02** | **3.55** | 7.85 | 11.01 | 8.67 | 11.97 | **0.00** | **2.21** | **4.01** | **1.22** |
| *Urocyon littoralis^!5^* | **0.00** | **1.18** | **1.73** | **1.63** | **1.83** | **2.85** | **2.05** | **3.32** | **1.97** | **2.24** | **3.91** | **1.44** |
| *Urocyon cinereoargenteus^16^* | **0.18** | **1.35** | **0.61** | **0.95** | **1.98** | **3.15** | **2.26** | **3.46** | **0.67** | **0.00** | **1.99** | **0.07** |
| *Urocyon cinereoargenteus^17^* | 42.28 | 57.57 | 6.47 | 8.49 | 36.73 | 54.91 | 45.94 | 61.75 | **1.06** | **1.30** | **2.19** | **0.00** |
| *Alopex lagopus^18^* | 56.96 | 35.90 | **3.31** | **5.81** | 59.62 | 38.24 | 56.07 | 35.50 | 34.28 | **3.60** | **1.85** | **0.00** |
| *Alopex lagopus^19^* | 6.59 | 8.62 | **1.76** | **1.95** | 7.98 | 9.65 | 8.90 | 11.01 | **1.13** | **0.00** | **2.29** | **0.53** |
| *Canis lupus^20^* | **0.00** | **0.91** | **1.25** | **1.32** | **1.79** | **2.64** | **2.10** | **3.08** | **1.54** | **1.50** | **2.59** | **0.88** |
| *Canis lupus^21^* | **2.94** | **1.37** | **4.80** | **3.22** | **5.08** | **3.27** | **4.89** | **3.44** | **0.99** | 9.90 | **0.00** | **4.49** |
| *Lycaon pictus^22^* | 26.15 | 17.42 | 26.83 | 19.42 | 29.46 | 19.66 | 27.52 | 19.55 | **0.00** | 86.84 | 11.70 | 36.18 |
| *Lycaon pictus^23^* | 31.52 | 23.62 | **0.35** | **0.00** | 16.94 | 25.82 | 32.00 | 24.20 | **3.54** | **0.63** | 404.40 | 52.42 |
| *Lycaon pictus^23^* | **0.00** | **0.00** | **2.00** | **1.77** | **2.03** | **1.82** | **2.02** | **2.14** | **1.20** | **5.11** | **0.27** | **1.52** |
| *Lycaon pictus^23^* | 13.35 | 8.03 | **4.31** | **2.76** | 15.43 | 10.12 | 14.72 | 9.48 | **0.00** | 7.12 | 36.79 | 38.13 |
| *Nyctereutes procyonoides^24^* | 6.83 | 7.91 | **3.30** | **3.09** | 8.29 | 9.08 | 9.15 | 10.30 | **3.65** | 7.65 | **0.00** | **4.67** |
| *Procyon lotor^25^* | 8.02 | 12.44 | **0.90** | **3.28** | 8.84 | 13.44 | 10.29 | 14.80 | **0.00** | **0.33** | **2.28** | **0.06** |
| *Crocuta crocuta^26^* | 20.19 | 25.14 | 6.93 | 12.12 | 20.38 | 25.99 | 22.63 | 27.67 | **0.00** | **0.00** | **2.00** | **0.00** |
| *Crocuta crocuta^26^* | 15.11 | 20.62 | **1.80** | **5.03** | 14.55 | 20.71 | 17.42 | 23.02 | **0.00** | **0.01** | **2.01** | **0.00** |
| *Crocuta crocuta^27^* | **2.34** | **3.96** | **2.51** | **3.01** | **4.07** | **5.66** | **4.46** | 6.12 | **2.62** | **0.00** | **2.51** | **1.12** |
| *Acinonyx jubatus^28^* | 10.76 | 14.70 | **3.63** | **4.71** | 11.97 | 15.80 | 13.18 | 17.25 | **0.26** | **0.59** | **2.29** | **0.00** |
| *Felis concolor^29^* | **5.80** | 9.26 | **1.10** | **2.44** | 7.01 | 10.51 | 8.05 | 11.60 | **0.00** | **3.16** | **4.87** | **1.77** |
| *Felis concolor^30^* | 8.71 | 12.77 | **2.50** | **4.82** | 9.81 | 13.72 | 11.04 | 15.21 | **0.00** | **2.29** | **4.11** | **0.67** |
| *Felis concolor^31^* | 68.08 | 108.76 | 7.17 | 19.71 | 61.30 | 100.73 | 72.28 | 113.89 | **0.50** | **3.78** | **3.87** | **0.00** |
| *Felis iriomotensis^32^* | 9.54 | 12.73 | **5.21** | 6.63 | 10.92 | 14.13 | 11.78 | 15.05 | **1.33** | **0.00** | **2.22** | **0.59** |
| *Lynx pardinus^33^* | **0.00** | **0.10** | **1.87** | **1.17** | **1.94** | **1.94** | **2.03** | **2.19** | **0.36** | **3.24** | **4.35** | **1.63** |
| *Panthera tigris altaica^34^* | **0.00** | **0.66** | **1.43** | **1.22** | **1.78** | **2.44** | **2.04** | **2.73** | **1.24** | **1.94** | **3.85** | **1.41** |
| *Panthera onca^35^* | **3.00** | 6.92 | 17.26 | 6.52 | 7.71 | 13.47 | **5.00** | 8.92 | **2.34** | **0.00** | **2.54** | **0.44** |
| *Panthera leo^36^* | 13.25 | 18.61 | **3.49** | 18.69 | 19.19 | 28.67 | 15.25 | 20.61 | **0.00** | **1.27** | **2.46** | **0.46** |
| *Panthera leo^37^* | 12.79 | 19.25 | **4.45** | 15.70 | 18.24 | 30.54 | 14.79 | 21.25 | **0.00** | **2.09** | **2.71** | **0.71** |
| *Panthera leo^37^* | 7.44 | 16.28 | 21.04 | 13.55 | 15.09 | 33.57 | 9.44 | 18.28 | **0.00** | 8.59 | **5.55** | **4.40** |
| *Panthera leo^38^* | 9.91 | 27.17 | 333.42 | 12.03 | 18.61 | 51.26 | 11.91 | 29.17 | **3.29** | **2.72** | **2.00** | **0.00** |
| *Panthera leo^38^* | **3.14** | 14.03 | 168.02 | 38.55 | 10.20 | 32.22 | **5.14** | 16.04 | **0.00** | **4.15** | **2.07** | **0.07** |
| *Panthera leo^38^* | **0.00** | **0.21** | **1.27** | **4.61** | **1.97** | **2.12** | **2.02** | **2.24** | **1.25** | **1.25** | **3.25** | **1.25** |
| *Panthera pardus^39^* | **0.00** | **0.13** | **1.60** | **2.21** | **1.89** | **2.03** | **2.01** | **2.14** | **1.61** | **1.61** | **3.60** | **1.60** |
| *Leopardus pardalis^40^* | 6.20 | 7.79 | **3.93** | **5.58** | 7.81 | 9.46 | 8.35 | 9.96 | **0.00** | **0.00** | **2.00** | **0.00** |
| *Ursus maritimus^41^* | 104.86 | 140.98 | 30.90 | 57.46 | 98.59 | 134.43 | 109.25 | 145.54 | **0.00** | **2.22** | **3.93** | **0.43** |
| *Ursus maritimus^42^* | 38.78 | 46.84 | 21.48 | 29.32 | 38.85 | 47.12 | 41.29 | 49.45 | **0.00** | **0.00** | **2.00** | **0.00** |
| *Ursus maritimus^42^* | 10.08 | 13.35 | 6.87 | 9.07 | 11.53 | 14.81 | 12.26 | 15.57 | **2.88** | **0.00** | **2.13** | **0.94** |
| *Ursus maritimus^42^* | 45.17 | 54.17 | 23.05 | 32.42 | 44.49 | 54.24 | 48.00 | 57.16 | **0.00** | **0.00** | **2.00** | **0.00** |
| *Ursus arctos^43^* | 21.56 | 29.47 | 7.02 | 10.95 | 21.81 | 29.64 | 24.16 | 32.26 | **0.00** | 6.37 | 7.93 | **3.69** |
| *Ursus arctos^43^* | 12.29 | 18.64 | **1.91** | **5.28** | 12.84 | 19.36 | 14.77 | 21.29 | **0.00** | **2.92** | **4.66** | **0.80** |
| *Ursus arctos^43^* | 25.46 | 37.80 | **4.78** | 11.51 | 24.78 | 37.01 | 28.15 | 40.73 | **0.00** | **2.59** | **4.28** | **1.16** |
| *Ursus arctos^44^* | 20.42 | 30.07 | 6.18 | 11.24 | 20.67 | 30.38 | 23.02 | 32.87 | **1.37** | **0.26** | **2.09** | **0.00** |
| *Ursus americanus^45^* | 6.36 | 9.36 | **3.65** | **5.29** | 7.76 | 10.86 | 8.55 | 11.61 | **1.92** | **0.00** | **2.01** | **0.46** |
| *Ursus americanus^46^* | 23.92 | 35.60 | **3.74** | 8.60 | 24.16 | 34.60 | 26.73 | 38.70 | **0.00** | 8.70 | 9.92 | **4.76** |
| *Ursus americanus^47^* | **2.50** | **4.36** | **0.83** | **1.48** | **4.07** | **5.91** | **4.66** | 6.57 | **0.00** | **1.63** | **3.43** | **0.77** |
| *Ursus americanus^48^* | **0.86** | **2.41** | **0.01** | **0.55** | **2.36** | **4.03** | **2.95** | **4.54** | **0.00** | **0.23** | **2.20** | **0.05** |
| *Ursus americanus^49^* | 18.86 | 28.04 | **5.34** | 10.04 | 17.18 | 27.85 | 21.44 | 30.82 | **1.03** | **0.45** | **2.25** | **0.00** |
| *Ursus americanus^50^* | 35.18 | 51.60 | 8.25 | 15.24 | 34.02 | 49.15 | 38.11 | 54.87 | **0.00** | 6.64 | 8.35 | **4.89** |
| *Ursus americanus^51^* | 20.03 | 26.62 | 7.87 | 11.09 | 20.42 | 26.98 | 22.58 | 29.33 | **0.00** | **5.01** | 6.64 | **2.85** |
| *Lutra lutra^52^* | 20.88 | 40.02 | **3.58** | 7.70 | 15.13 | 38.62 | 23.85 | 43.47 | **5.38** | **0.88** | **2.23** | **0.00** |
| *Lutra lutra^53^* | **2.04** | **3.97** | **0.32** | **1.21** | **3.56** | **5.53** | **4.19** | 6.17 | **0.00** | **1.19** | **3.08** | **0.33** |
| *Lutra lutra^54^* | **0.00** | **1.25** | **1.44** | **1.87** | **1.71** | **2.92** | **2.05** | **3.33** | **2.05** | **0.58** | **2.66** | **1.04** |
| *Lutra lutra^55^* | **3.66** | 7.32 | **0.19** | **1.38** | **4.84** | 8.67 | **5.89** | 9.64 | **0.00** | **0.80** | **2.61** | **0.14** |
| *Lutra lutra^55^* | 12.39 | 25.20 | **0.02** | **1.75** | 11.83 | 24.68 | 15.07 | 28.22 | **0.00** | **3.35** | **4.42** | **1.12** |
| *Lutra lutra^55^* | **2.43** | 6.44 | **0.87** | **1.65** | **3.61** | 7.62 | **4.63** | 8.75 | **1.47** | **0.00** | **1.90** | **0.05** |
| *Lontra canadensis^56^* | **0.25** | **1.29** | **0.20** | **0.67** | **2.01** | **3.08** | **2.33** | **3.40** | **0.00** | **0.77** | **2.71** | **0.23** |
| *Mustela erminea^57^* | **2.28** | **3.24** | **0.16** | **0.20** | **3.90** | **4.81** | **4.51** | **5.52** | 18.60 | **0.18** | **2.01** | **0.00** |
| *Mustela nigripes^58^* | 27.40 | 39.28 | **5.59** | 9.16 | 26.56 | 37.76 | 30.42 | 42.64 | **0.00** | **4.72** | **5.31** | **1.48** |
| *Martes pennanti^59^* | 12.02 | 14.17 | 8.77 | 9.76 | 13.59 | 15.75 | 14.26 | 16.47 | **0.01** | **0.00** | **2.00** | **0.00** |
| *Martes americana^60^* | **0.00** | **0.47** | **0.34** | **0.74** | **1.78** | **2.33** | **2.04** | **2.52** | **0.13** | **0.13** | **2.13** | **0.13** |
| *Spilogale putorius^61^* | **0.08** | **1.04** | **0.45** | **0.02** | **1.84** | **2.69** | **2.20** | **3.23** | **0.00** | **2.90** | **2.98** | **1.19** |
| *Gulo gulo^62^* | 7.35 | 11.60 | **1.60** | **3.44** | 8.41 | 12.27 | 9.65 | 14.01 | **0.00** | **2.06** | **3.82** | **0.96** |
| *Meles meles^63^* | 9.26 | 14.84 | **1.36** | **2.62** | 10.05 | 15.64 | 11.68 | 17.43 | **0.00** | **4.38** | **3.31** | **2.61** |
| *Meles meles^63^* | 24.48 | 40.10 | 7.65 | 13.23 | 23.92 | 39.50 | 27.23 | 43.15 | 6.56 | **0.00** | **2.12** | **0.89** |
| **Frequency of**  **ΔAIC = 0** | 0.13 | 0.00 | 0.04 | 0.03 | 0.00 | 0.00 | 0.00 | 0.00 | 0.47 | 0.15 | 0.03 | 0.15 |
| **Frequency of**  **ΔAIC ≤ 6** | 0.37 | 0.26 | 0.73 | 0.63 | 0.31 | 0.26 | 0.32 | 0.22 | 0.95 | 0.77 | 0.86 | 0.87 |
