# Supplementary material for: Does Litter Size Variation Affect Models of Terrestrial Carnivore Extinction Risk and Management?
Source: PLoS One. 2013 Feb 28;8(2):e58060. doi: 10.1371/journal.pone.0058060 (PMC3585178; doi:10.1371/journal.pone.0058060)
Supplement: Table S3 — Results of the Fisher Exact test goodness-of-fit of probability distributions to empirical carnivore litter size frequencies. (DOC) [file pone.0058060.s003.doc]

Table S3. Results of the Fisher Exact test goodness-of-fit of probability distributions to empirical carnivore litter size frequencies. Distributions with *p* < 0.05 were classified as not fitting. Bold indicates the distributions for which model selection determined ΔAIC ≤ 6. References refer to those in Table S1. Distribution abbreviations: SP: Shifted Poisson; ZTP: Zero-truncated Poisson; SB: Shifted binomial; ZTB: Zero-truncated binomial; SNB: Shifted negative binomial; ZTNB: Zero-truncated negative binomial; SGP: Shifted generalised Poisson; ZTGP: Zero-truncated generalised Poisson; DN: Discretised normal; DLN: Discretised lognormal; DSB3; Discretised stretched-beta (3 parameter form); DSB2; Discretised stretched-beta (2 parameter form).

|  | ***Distribution*** | | | | | | | | | | | |
| --- | --- | --- | --- | --- | --- | --- | --- | --- | --- | --- | --- | --- |
| ***Species^(reference)^*** | ***SP*** | ***ZTP*** | ***SB*** | ***ZTB*** | ***SNB*** | ***ZTNB*** | ***SGP*** | ***ZTGP*** | ***DN*** | ***DLN*** | ***DSB3*** | ***DSB2*** |
| *Vulpes velox^1^* | **0.846** | 0.837 | **0.991** | **0.990** | 0.875 | 0.859 | 0.853 | 0.824 | **0.811** | **0.764** | **0.713** | **0.795** |
| *Vulpes macrotis^2^* | 0.205 | 0.025 | **0.693** | **0.885** | 0.268 | 0.059 | 0.162 | 0.019 | **0.914** | 0.148 | **0.841** | 0.409 |
| *Vulpes macrotis^3^* | 0.132 | 0.093 | 0.361 | 0.354 | 0.145 | 0.086 | 0.149 | 0.100 | **1.000** | **1.000** | **1.000** | **1.000** |
| *Vulpes vulpes^4^* | 0.018 | 0.001 | **0.982** | **0.994** | 0.038 | 0.003 | 0.009 | 0.002 | **0.996** | 0.430 | **0.955** | 0.729 |
| *Vulpes vulpes^5^* | **0.238** | 0.084 | **0.274** | **0.359** | **0.271** | 0.123 | **0.199** | 0.073 | **0.253** | 0.119 | **0.147** | **0.094** |
| *Vulpes vulpes^6^* | **0.652** | **0.662** | **0.644** | **0.611** | **0.623** | **0.699** | **0.748** | **0.699** | **0.353** | 0.538 | **0.637** | **0.634** |
| *Vulpes vulpes^7^* | 0.246 | 0.058 | **0.704** | **0.819** | 0.299 | 0.146 | 0.242 | 0.054 | **0.844** | 0.104 | **0.633** | 0.321 |
| *Vulpes vulpes^8^* | 0.004 | 0.154 | 0.182 | 0.344 | 0.004 | 0.098 | 0.007 | 0.160 | **0.777** | 0.086 | **0.419** | 0.260 |
| *Vulpes vulpes^8^* | **0.538** | 0.320 | **0.580** | **0.662** | **0.630** | 0.431 | 0.565 | 0.300 | **0.705** | **0.555** | **0.394** | **0.522** |
| *Vulpes vulpes^8^* | **0.504** | 0.282 | **0.712** | **0.810** | 0.598 | 0.391 | 0.542 | 0.253 | **0.923** | 0.164 | 0.478 | 0.386 |
| *Vulpes vulpes^9^* | 0.031 | 0.003 | **0.184** | **0.176** | 0.066 | 0.013 | 0.030 | 0.005 | **0.283** | 0.194 | 0.157 | **0.096** |
| *Vulpes vulpes^10^* | 0.818 | 0.515 | **0.992** | **0.993** | 0.838 | 0.595 | 0.787 | 0.527 | **0.986** | **0.904** | **0.994** | **0.968** |
| *Vulpes vulpes^11^* | 0.006 | 0.000 | **0.604** | **0.673** | 0.033 | 0.000 | 0.005 | 0.000 | **0.704** | 0.072 | 0.371 | 0.162 |
| *Vulpes vulpes^12^* | **0.209** | **0.392** | **0.190** | **0.282** | **0.147** | **0.384** | **0.207** | **0.403** | **0.379** | 0.124 | **0.211** | **0.215** |
| *Vulpes vulpes^13^* | 0.000 | 0.000 | 0.000 | 0.000 | 0.000 | 0.000 | 0.000 | 0.000 | 0.000 | **0.050** | 0.000 | 0.000 |
| *Urocyon littoralis^14^* | 0.301 | 0.179 | **0.717** | **0.599** | 0.352 | 0.167 | 0.299 | 0.157 | **0.849** | **0.517** | **0.528** | **0.577** |
| *Urocyon littoralis^!5^* | **0.742** | **0.739** | **0.733** | **0.672** | **0.766** | **0.752** | **0.727** | **0.736** | **0.600** | **0.729** | **0.758** | **0.749** |
| *Urocyon cinereoargenteus^16^* | **0.939** | **0.854** | **0.848** | **0.925** | **0.942** | **0.872** | **0.940** | **0.852** | **0.728** | **0.773** | **0.825** | **0.776** |
| *Urocyon cinereoargenteus^17^* | 0.001 | 0.000 | 0.543 | 0.448 | 0.000 | 0.000 | 0.000 | 0.000 | **0.713** | **0.802** | **0.792** | **0.719** |
| *Alopex lagopus^18^* | 0.000 | 0.001 | **0.698** | **0.505** | 0.000 | 0.003 | 0.000 | 0.001 | 0.001 | **0.778** | **0.799** | **0.601** |
| *Alopex lagopus^19^* | 0.721 | 0.669 | **0.630** | **0.558** | 0.739 | 0.694 | 0.715 | 0.662 | **0.585** | **0.588** | **0.633** | **0.575** |
| *Canis lupus^20^* | **0.920** | **0.949** | **0.742** | **0.772** | **0.915** | **0.952** | **0.936** | **0.946** | **0.748** | **0.742** | **0.843** | **0.789** |
| *Canis lupus^21^* | **0.059** | 0.146 | **0.081** | **0.092** | **0.044** | **0.104** | **0.060** | **0.153** | **0.183** | 0.086 | **0.188** | **0.117** |
| *Lycaon pictus^22^* | 0.000 | 0.000 | 0.000 | 0.000 | 0.000 | 0.000 | 0.000 | 0.001 | **0.001** | 0.000 | 0.000 | 0.000 |
| *Lycaon pictus^23^* | 0.001 | 0.002 | **0.926** | **0.945** | 0.449 | 0.001 | 0.001 | 0.002 | **0.521** | **0.958** | 0.000 | 0.000 |
| *Lycaon pictus^23^* | **0.893** | 0.937 | **0.868** | **0.895** | **0.847** | **0.904** | **0.882** | **0.960** | **0.936** | **0.836** | **0.977** | **0.899** |
| *Lycaon pictus^23^* | 0.003 | 0.007 | **0.224** | **0.255** | 0.002 | 0.006 | 0.002 | 0.004 | **0.201** | 0.248 | 0.000 | 0.000 |
| *Nyctereutes procyonoides^24^* | 0.725 | 0.733 | **0.363** | **0.398** | 0.707 | 0.735 | 0.689 | 0.737 | **0.452** | 0.314 | **0.921** | **0.391** |
| *Procyon lotor^25^* | 0.137 | 0.036 | **0.836** | **0.429** | 0.177 | 0.048 | 0.127 | 0.053 | **1.000** | **0.955** | **0.957** | **1.000** |
| *Crocuta crocuta^26^* | 0.004 | 0.000 | 0.465 | 0.025 | 0.001 | 0.002 | 0.002 | 0.001 | **1.000** | **1.000** | **1.000** | **1.000** |
| *Crocuta crocuta^26^* | 0.007 | 0.003 | **0.906** | **0.301** | 0.015 | 0.002 | 0.007 | 0.000 | **1.000** | **1.000** | **1.000** | **1.000** |
| *Crocuta crocuta^27^* | **0.577** | 0.513 | **0.413** | **0.375** | **0.594** | **0.478** | **0.568** | 0.518 | **0.296** | **0.473** | **0.437** | **0.401** |
| *Acinonyx jubatus^28^* | 0.387 | 0.175 | **0.809** | **0.759** | 0.352 | 0.199 | 0.369 | 0.171 | **0.762** | **0.767** | **0.788** | **0.805** |
| *Felis concolor^29^* | 0.300 | 0.140 | **0.672** | **0.600** | 0.367 | 0.155 | 0.279 | 0.128 | **0.739** | **0.433** | **0.395** | **0.492** |
| *Felis concolor^30^* | 0.124 | 0.061 | **0.567** | **0.403** | 0.167 | 0.068 | 0.151 | 0.053 | **0.828** | **0.528** | **0.517** | **0.668** |
| *Felis concolor^31^* | 0.000 | 0.000 | 0.358 | 0.035 | 0.000 | 0.000 | 0.000 | 0.000 | **0.843** | **0.722** | **0.868** | **0.935** |
| *Felis iriomotensis^32^* | 0.210 | 0.104 | **0.384** | 0.335 | 0.205 | 0.124 | 0.192 | 0.097 | **0.366** | **0.542** | **0.473** | **0.408** |
| *Lynx pardinus^33^* | 0.610 | 0.739 | **0.532** | **0.608** | **0.619** | **0.711** | **0.669** | **0.725** | **0.799** | **0.584** | **0.671** | **0.628** |
| *Panthera tigris altaica^34^* | 0.920 | 0.885 | **0.763** | **0.820** | **0.903** | **0.880** | **0.887** | **0.907** | **0.919** | **0.795** | **0.839** | **0.874** |
| *Panthera onca^35^* | 0.519 | 0.255 | 0.605 | 0.446 | 0.508 | 0.269 | **0.535** | 0.230 | **0.432** | **0.927** | **0.923** | **0.822** |
| *Panthera leo^36^* | 0.080 | 0.019 | **0.614** | 0.392 | 0.109 | 0.035 | 0.110 | 0.023 | **1.000** | **0.792** | **0.785** | **0.866** |
| *Panthera leo^37^* | 0.091 | 0.013 | **0.615** | 0.440 | 0.092 | 0.026 | 0.089 | 0.013 | **0.788** | **0.542** | **0.530** | **0.616** |
| *Panthera leo^37^* | 0.407 | 0.074 | 0.852 | 0.950 | 0.508 | 0.089 | 0.373 | 0.056 | **0.957** | 0.432 | **0.505** | **0.690** |
| *Panthera leo^38^* | 0.177 | 0.003 | 0.794 | 0.641 | 0.282 | 0.017 | 0.156 | 0.001 | **0.520** | **0.750** | **0.713** | **0.841** |
| *Panthera leo^38^* | 0.371 | 0.044 | 0.761 | 0.634 | 0.532 | 0.076 | **0.363** | 0.041 | **0.769** | **0.518** | **0.610** | **0.490** |
| *Panthera leo^38^* | 0.794 | 0.799 | **1.000** | **0.726** | **1.000** | **0.803** | **0.796** | **0.808** | **1.000** | **1.000** | **1.000** | **1.000** |
| *Panthera pardus^39^* | 0.814 | 0.808 | **1.000** | **0.821** | **1.000** | **1.000** | **0.800** | **0.816** | **1.000** | **1.000** | **1.000** | **1.000** |
| *Leopardus pardalis^40^* | 0.169 | 0.153 | **0.351** | **0.152** | 0.162 | 0.123 | 0.169 | 0.141 | **1.000** | **1.000** | **1.000** | **1.000** |
| *Ursus maritimus^41^* | 0.000 | 0.000 | 0.001 | 0.000 | 0.000 | 0.000 | 0.000 | 0.000 | **1.000** | **0.948** | **0.948** | **0.988** |
| *Ursus maritimus^42^* | 0.000 | 0.000 | 0.000 | 0.000 | 0.000 | 0.000 | 0.000 | 0.000 | **1.000** | **1.000** | **1.000** | **1.000** |
| *Ursus maritimus^42^* | 0.035 | 0.008 | 0.034 | 0.037 | 0.026 | 0.011 | 0.031 | 0.010 | **0.069** | **0.225** | **0.215** | **0.139** |
| *Ursus maritimus^42^* | 0.000 | 0.000 | 0.000 | 0.000 | 0.000 | 0.000 | 0.000 | 0.000 | **1.000** | **1.000** | **1.000** | **1.000** |
| *Ursus arctos^43^* | 0.002 | 0.001 | 0.137 | 0.071 | 0.002 | 0.001 | 0.002 | 0.000 | **0.418** | 0.061 | 0.068 | **0.134** |
| *Ursus arctos^43^* | 0.049 | 0.013 | **0.643** | **0.247** | 0.058 | 0.010 | 0.042 | 0.006 | **0.853** | **0.511** | **0.543** | **0.710** |
| *Ursus arctos^43^* | 0.001 | 0.000 | **0.411** | 0.046 | 0.002 | 0.000 | 0.001 | 0.000 | **0.997** | **0.748** | **0.765** | **0.876** |
| *Ursus arctos^44^* | 0.009 | 0.000 | 0.284 | 0.103 | 0.014 | 0.000 | 0.006 | 0.001 | **0.619** | **0.975** | **0.983** | **0.952** |
| *Ursus americanus^45^* | 0.220 | 0.117 | **0.366** | **0.277** | 0.230 | 0.118 | 0.218 | 0.105 | **0.250** | **0.541** | **0.551** | **0.405** |
| *Ursus americanus^46^* | 0.002 | 0.000 | **0.270** | 0.130 | 0.003 | 0.000 | 0.002 | 0.000 | **0.570** | 0.066 | 0.081 | **0.157** |
| *Ursus americanus^47^* | 0.727 | **0.568** | **0.946** | **0.908** | 0.737 | **0.578** | **0.702** | 0.550 | **0.840** | **0.611** | **0.676** | **0.677** |
| *Ursus americanus^48^* | 0.645 | **0.501** | **1.000** | **0.910** | 0.685 | **0.493** | **0.639** | **0.506** | **1.000** | **0.912** | **0.907** | **1.000** |
| *Ursus americanus^49^* | 0.013 | 0.001 | **0.382** | 0.146 | 0.024 | 0.002 | 0.011 | 0.001 | **0.717** | **0.961** | **0.981** | **0.965** |
| *Ursus americanus^50^* | 0.000 | 0.000 | 0.001 | 0.000 | 0.000 | 0.000 | 0.000 | 0.000 | **0.016** | 0.000 | 0.001 | **0.001** |
| *Ursus americanus^51^* | 0.005 | 0.001 | 0.141 | 0.076 | 0.003 | 0.001 | 0.007 | 0.001 | **0.412** | **0.092** | 0.126 | **0.220** |
| *Lutra lutra^52^* | 0.627 | **0.456** | **0.905** | **0.843** | 0.654 | **0.446** | **0.612** | 0.444 | **0.857** | **0.752** | **0.770** | **0.822** |
| *Lutra lutra^53^* | 0.425 | **0.346** | **0.312** | **0.311** | **0.438** | **0.357** | **0.429** | **0.382** | **0.272** | **0.485** | **0.481** | **0.424** |
| *Lutra lutra^54^* | 0.609 | 0.317 | **0.993** | **0.887** | **0.653** | 0.363 | **0.620** | 0.285 | **0.982** | **0.919** | **0.919** | **0.987** |
| *Lutra lutra^55^* | 0.120 | 0.007 | **0.962** | **0.771** | 0.210 | 0.008 | 0.090 | 0.004 | **0.936** | **0.775** | **0.834** | **0.848** |
| *Lutra lutra^55^* | 0.445 | 0.213 | **0.569** | **0.499** | **0.544** | 0.233 | **0.445** | 0.223 | **0.389** | **0.791** | **0.827** | **0.711** |
| *Lutra lutra^55^* | 0.748 | **0.686** | **0.933** | **0.896** | **0.753** | **0.677** | **0.730** | **0.679** | **0.862** | **0.764** | **0.756** | **0.795** |
| *Lontra canadensis^56^* | 0.005 | 0.000 | **0.699** | 0.411 | 0.009 | 0.000 | 0.005 | 0.000 | **0.969** | **0.542** | **0.692** | **0.800** |
| *Mustela erminea^57^* | 0.225 | 0.136 | 0.437 | 0.396 | 0.250 | 0.142 | 0.230 | 0.145 | **1.000** | **1.000** | **1.000** | **1.000** |
| *Mustela nigripes^58^* | 0.727 | **0.647** | **1.000** | **0.759** | **0.699** | **0.659** | **0.712** | **0.672** | **1.000** | **1.000** | **1.000** | **1.000** |
| *Martes pennanti^59^* | **0.674** | **0.678** | **0.473** | **0.521** | **0.660** | **0.713** | **0.652** | **0.690** | **0.601** | **0.460** | **0.567** | **0.468** |
| *Martes americana^60^* | 0.344 | 0.145 | **0.871** | **0.713** | 0.345 | 0.153 | 0.311 | 0.143 | **0.966** | **0.752** | **0.734** | **0.820** |
| *Spilogale putorius^61^* | 0.175 | 0.071 | **0.564** | **0.579** | 0.225 | 0.060 | 0.163 | 0.052 | **0.665** | **0.322** | **0.403** | **0.391** |
| *Gulo gulo^62^* | 0.002 | 0.000 | 0.142 | 0.028 | 0.004 | 0.000 | 0.005 | 0.000 | 0.207 | **0.983** | **0.934** | **0.892** |
| *Meles meles^63^* | **0.846** | 0.837 | **0.991** | **0.990** | 0.875 | 0.859 | 0.853 | 0.824 | **0.811** | **0.764** | **0.713** | **0.795** |
| *Meles meles^63^* | 0.205 | 0.025 | **0.693** | **0.885** | 0.268 | 0.059 | 0.162 | 0.019 | **0.914** | 0.148 | **0.841** | 0.409 |
