# Supplementary material for: Does Litter Size Variation Affect Models of Terrestrial Carnivore Extinction Risk and Management?
Source: PLoS One. 2013 Feb 28;8(2):e58060. doi: 10.1371/journal.pone.0058060 (PMC3585178; doi:10.1371/journal.pone.0058060)
Supplement: Table S4 — Coefficient of variation for model outcomes of quasi-extinction probabilities and probability of successful disease control, for 12 probability distributions. (DOC) [file pone.0058060.s004.doc]

Table S4. Coefficient of variation for model outcomes of quasi-extinction probabilities* and probability of successful disease control^†^, for 12 probability distributions. Distribution^§^ abbreviations: SP: Shifted Poisson; ZTP: Zero-truncated Poisson; SB: Shifted binomial; ZTB: Zero-truncated binomial; SNB: Shifted negative binomial; ZTNB: Zero-truncated negative binomial; SGP: Shifted generalised Poisson; ZTGP: Zero-truncated generalised Poisson; DN: Discretised normal; DLN: Discretised lognormal; DSB3; Discretised stretched-beta (3 parameter form); DSB2; Discretised stretched-beta (2 parameter form).

| **Distribution^§^** | **Island fox* (West)** | **Island fox* (East)** | **Red fox^†^** | **African wild dog without Allee*** | **African wild dog with Allee*** |
| --- | --- | --- | --- | --- | --- |
| *SP* | 1.161 | 1.354 | 0.307 | 1.388 | 0.713 |
| *ZTP* | 1.116 | 1.298 | 0.333 | 1.407 | 0.703 |
| *SGP* | 1.102 | 1.302 | 0.332 | 1.409 | 0.709 |
| *ZTGP* | 1.130 | 1.339 | 0.332 | 1.416 | 0.712 |
| *SB* | 1.154 | 1.350 | 0.293 | 1.409 | 0.668 |
| *ZTB* | 1.126 | 1.335 | 0.306 | 1.418 | 0.689 |
| *SNB* | 1.134 | 1.330 | 0.332 | 1.431 | 0.712 |
| *ZTNB* | 1.122 | 1.307 | 0.332 | 1.428 | 0.736 |
| *DN* | 1.161 | 1.353 | 0.307 | 1.399 | 0.712 |
| *DLN* | 1.143 | 1.339 | 0.319 | 1.424 | 0.737 |
| *DSB2* | 1.133 | 1.336 | 0.307 | 1.403 | 0.711 |
| *DSB3* | 1.133 | 1.319 | 0.320 | 1.406 | 0.710 |
