# Supplementary material for: Does Litter Size Variation Affect Models of Terrestrial Carnivore Extinction Risk and Management?
Source: PLoS One. 2013 Feb 28;8(2):e58060. doi: 10.1371/journal.pone.0058060 (PMC3585178; doi:10.1371/journal.pone.0058060)
Supplement: Appendix S1 — Testing for intraspecific variation in litter size distributions, using the red fox Vulpes vulpes as an example. (DOC) [file pone.0058060.s005.doc]

Appendix S1. Testing for intraspecific variation in litter size distributions

In addition to establishing whether interspecific differences exist in the suitability of probability distributions to model litter size, it is also interesting to consider intraspecific variation in describing litter size. Intraspecific variation can be examined through a two-part analysis. We first sought evidence that distinct probability distributions are required to describe the litter size distributions of conspecific populations. Specifically, do we need distinct probability distributions to describe the litter size data (hereafter referred to as “a dataset”) taken from populations that are separated geographically, or where the data have been determined using different methods? Second, if it is established that a given distribution can in fact be adequately applied to multiple datasets, do the same parameter values of the specified probability distribution function describe these datasets adequately?

The first component of the analyses determined whether a given probability distribution adequately describes two or more datasets. For a specified pair of datasets, the joint AIC value was calculated for each possible probability distribution combination. Specifically, let *M*(*i*,*j*) be a model where probability distribution *i* is fitted to the first dataset and probability distribution *j* is fitted to the second dataset. The log-likelihood of this model is then simply the sum of the log-likelihoods of each probability distribution fitted to their specified dataset. We evaluated whether a given probability distributions adequately described the focal datasets by determining if any model where *i* = *j* was within 6 units of the smallest AIC (over all possible probability distribution combinations). This approach is readily generalised for more than two datasets. Only those distributions which the initial analysis (see *Methods: probability distribution fitting*, Table 2 and Table S2) identified as parsimonious (for the geographic and methodological datasets, respectively) were included in these analyses of intraspecific variation.

If at least one probability distribution could adequately describe all the focal datasets, the second component of the analyses sought to determine whether the same parameter values could be used to describe each of the datasets. Specifically, let$LL(\hat{\theta}|S)$be the maximum log-likelihood when the probability distribution described by the parameters **θ** is fitted to dataset *S*. The maximum log-likelihood when two datasets are described by distinct parameter sets is$LL_{1}=LL(\hat{\theta}_{1}|S_{1})+LL(\hat{\theta}_{2}|S_{2})$; and when the two datasets are described by a probability distribution with the same parameters, the maximum log-likelihood is$LL_{0}=LL(\hat{\theta}_{3}|S_{1})+LL(\hat{\theta}_{3}|S_{2})$. A log-likelihood ratio test was then used to determine whether the simpler model (using a single parameter set) provided a more parsimonious description of the combined datasets than its expanded alternative (using two distinct parameter sets). The test statistic is determined by the deviance, defined as *G*=2(*LL*_1_-*LL*_0_). The distribution of *G* is approximately chi-squared, with the degrees of freedom (df) equal to the additional number of free parameters required for the more complex model . This approach is also readily generalised for more than two datasets.

We used the above approaches to test for intraspecific differences in the underlying litter size distributions of the red fox *Vulpes vulpes*. We used litter size data collected from six geographically distinct populations, where data were determined by placental scars. Data for these populations were combined over 4, 3, 4, 5, 6, and 17 year periods, respectively (Table S1). We then compared three methods used to determine litter size for one *V. vulpes* population [2], using data determined by placental scars, embryo counts and direct counts, combined over a 17 year period.

Model selection results for the specified geographically distinct *V. vulpes* populations supported models using the same distribution (Table A1), suggesting that the focal datasets could be described adequately using the discretised normal. Further, a single parameter set adequately described the discretised normal litter size distribution (*G* = 119.23, df = 10, *p* <0.001) for these geographically separated *V. vulpes* populations. For litter size data of a *V. vulpes* population determined by different methods, a difference in the underlying distributions was inferred by the lack of support for models using the same distributions (Table A2). Thus, these methodological datasets were best described by distinct distributions and parameter sets.

Table A1. Results of model selection to test for intraspecific geographic variation in the best-fitting litter size distributions for *V. vulpes* populations. Only models where datasets fitted to probability distribution* combinations had a ∆AIC score ≤ 6 are presented. For details of the datasets, refer to the references in Table S1.

| ***Dataset ^[Reference]^*** | | | | | | ***Log- likelihood*** |  |  |
| --- | --- | --- | --- | --- | --- | --- | --- | --- |
| ***1^[4]^*** | ***2^[8]^*** | ***3^[8]^*** | ***4^[8]^*** | ***5^[9]^*** | ***6^[11]^*** |  | ***AIC*** | ***∆AIC*** |
| DN | DN | SP | DN | DN | DN | -118.20 | 260.41 | 5.69 |
| DN | DN | DN | DN | DN | DN | -115.36 | 254.72 | 0.00 |
| DN | DN | DN | SB | DN | DN | -117.32 | 258.64 | 3.92 |
| DN | DN | DN | ZTSB | DN | DN | -117.73 | 259.47 | 4.75 |
| DN | DN | DLN | DN | DN | DN | -118.08 | 260.17 | 5.45 |
| DN | DN | ZTSB | DN | DN | DN | -115.53 | 255.06 | 0.34 |
| DN | DN | ZTSB | SB | DN | DN | -117.49 | 258.98 | 4.26 |
| DN | DN | ZTSB | ZTSB | DN | DN | -117.91 | 259.81 | 5.09 |
| DN | DN | DSB3 | DN | DN | DN | -116.10 | 258.20 | 3.48 |
| DN | DN | DSB2 | DN | DN | DN | -116.92 | 257.84 | 3.12 |
| DN | DSB3 | DN | DN | DN | DN | -117.17 | 260.34 | 5.62 |
| DN | DSB3 | ZTSB | DN | DN | DN | -117.34 | 260.68 | 5.96 |
| SB | DN | SP | DN | DN | DN | -118.32 | 260.64 | 5.92 |
| SB | DN | DN | DN | DN | DN | -115.48 | 254.95 | 0.23 |
| SB | DN | DN | SB | DN | DN | -117.43 | 258.87 | 4.15 |
| SB | DN | DN | ZTSB | DN | DN | -117.85 | 259.70 | 4.98 |
| SB | DN | DLN | DN | DN | DN | -118.20 | 260.40 | 5.68 |
| SB | DN | ZTSB | DN | DN | DN | -115.65 | 255.30 | 0.58 |
| SB | DN | ZTSB | SB | DN | DN | -117.61 | 259.21 | 4.49 |
| SB | DN | ZTSB | ZTSB | DN | DN | -118.02 | 260.04 | 5.32 |
| SB | DN | DSB3 | DN | DN | DN | -116.22 | 258.43 | 3.71 |
| SB | DN | DSB2 | DN | DN | DN | -117.04 | 258.07 | 3.35 |
| SB | DSB3 | DN | DN | DN | DN | -117.28 | 260.57 | 5.85 |
| DSB3 | DN | DN | DN | DN | DN | -115.75 | 257.50 | 2.78 |
| DSB3 | DN | ZTSB | DN | DN | DN | -115.92 | 257.85 | 3.13 |
| DSB3 | DN | DSB2 | DN | DN | DN | -117.31 | 260.62 | 5.90 |

*Distribution abbreviations: SP: Shifted Poisson; ZTP: Zero-truncated Poisson; SB: Shifted binomial; ZTSB: Zero-truncated binomial; SNB: Shifted negative binomial; SGP: Shifted generalised Poisson; ZTGP: Zero-truncated generalised Poisson; DN: Discretised normal; DLN: Discretised lognormal; DSB3: Discretised stretched-beta (3 parameter form); DSB2: Discretised stretched-beta (2 parameter form).

Table A2. Results of model selection to test for intraspecific methodological variation in the best-fitting litter size distributions for a *V. vulpes* population. Only models where datasets fitted to probability distribution* combinations had a ∆AIC score ≤ 6 are presented. For details of the datasets, refer to the references in Table S1.

| ***Dataset^[Reference]^*** | | | ***Log- likelihood*** |  |  |
| --- | --- | --- | --- | --- | --- |
| ***1^[11]^*** | ***2^[12]^*** | ***3^[13]^*** |  | ***AIC*** | ***∆AIC*** |
| DN | SP | DLN | -83.63 | 177.27 | 2.24 |
| DN | ZTP | DLN | -83.20 | 176.40 | 1.38 |
| DN | DN | DLN | -81.51 | 175.02 | 0.00 |
| DN | SNB | DLN | -84.21 | 180.42 | 5.39 |
| DN | SGP | DLN | -83.63 | 179.27 | 4.24 |
| DN | ZTGP | DLN | -83.20 | 178.40 | 3.38 |
| DN | DSB2 | DLN | -83.66 | 179.31 | 4.29 |

**References**

1. Sokal RR, Rohlf FJ (1987) Introduction to biostatistics. New York: Freeman.

2. Harris, S. unpublished data.
