# Supplementary material for: Does Litter Size Variation Affect Models of Terrestrial Carnivore Extinction Risk and Management?
Source: PLoS One. 2013 Feb 28;8(2):e58060. doi: 10.1371/journal.pone.0058060 (PMC3585178; doi:10.1371/journal.pone.0058060)
Supplement: Appendix S2 — Functional forms for the 12 probability distributions fitted to empirical litter size frequency data. (DOC) [file pone.0058060.s006.doc]

Appendix S2. Functional forms for litter size probability distributions

The 12 probability distributions, *f*(*x*), fitted to the empirical litter size frequencies are described below. Here, *x* is the litter size, *y* is (*x*-1) and *x*_max_ is the maximum litter size for a given population. Γ is the complete gamma function and *λ*, *s* and *f_max_* are the parameters of the distributions fitted by maximum likelihood. Continuous distributions* were converted into discrete forms by calculating values for *x* = 1,2,…*x_max_* and rescaling the probabilities to sum to unity.

| ***Distribution*** | ***Functional form*** | ***Estimated parameters and possible range*** | ***Possible range of x*** |
| --- | --- | --- | --- |
| Shifted Poisson | $f(x\vert\lambda)=\frac{\lambda^{x}e^{-\lambda}}{x!}$ | λ > 0 | *x* ≥ 0 |
| Zero-truncated Poisson | $f(y\vert\lambda)=\frac{\lambda^{y}e^{-\lambda}}{(1-e^{-\lambda})y!}$ | λ > 0 | *y* ≥ 1 |
| Shifted Generalised Poisson^†^ | $f(x\vert\lambda,s)=\frac{\lambda(\lambda+xs)^{(x-1)}e^{-(\lambda+xs)}}{x!}$ | λ > 0 | *x* ≥ 0 |
| Zero-truncated Generalised Poisson^†^ | $f(y\vert\lambda,s)=\frac{\lambda(\lambda+ys)^{(y-1)}e^{-(\lambda+ys)}}{(1-e^{-\lambda}+ys)y!}$ | λ > 0 | *y* ≥ 1 |
| Shifted Binomial | $f(x\vert\lambda,s)=\frac{\lambda!}{(\lambda-x)!x!}s^{x}(1-s)^{(\lambda-x)}$ | λ is a positive integer,  0 ≤ *s* ≤ 1 | 0 ≤ *x* ≤ λ |
| Zero-truncated Binomial | $f(y\vert\lambda,s)=\frac{\lambda!}{(1-s)^{\lambda}(\lambda-y)!y!}s^{y}(1-s)^{(\lambda-y)}$ | λ is a positive integer,  0 ≤ *s* ≤ 1 | 1 ≤ *y* ≤ λ |
| Shifted Negative Binomial | $f(x\vert\lambda,s)=\frac{(x+\lambda-1)!}{(\lambda-1)!x!}s^{\lambda}(1-s)^{x}$ | λ > 0, *s* > 0 | 0 ≤ *x* ≤ ∞ |
| Zero-truncated Negative Binomial | $f(y\vert\lambda,s)=\frac{(y+\lambda-1)!}{(1-s)^{\lambda}(\lambda-1)!y!}s^{\lambda}(1-s)^{y}$ | λ > 0, *s* > 0 | 1 ≤ *y* ≤ ∞ |
| Discretised normal* | $f(x\vert\lambda,s)=\frac{1}{\sqrt{(2\pi)s^{2}}}e^{\frac{(x-\lambda)^{2}}{2s^{2}}}$ | *s* > 0 | 1 ≤ *x* ≤ *x*_max_ |
| Discretised lognormal* | $f(x\vert\lambda,s)=\frac{1}{\sqrt{(2\pi)sx}}e^{-\frac{(\ln x-\lambda)^{2}}{2s^{2}}}$ | λ > 0, *s* > 0 | 1 ≤ *x* ≤ *x*_max_ |
| Discretised stretched beta, 2 parameter form* | $f(z\vert\lambda,s)=\frac{\Gamma(a+b)}{\Gamma(a)\Gamma(b)}z^{(a-1)}(1-z)^{(b-1)}$,  where:  $z=\frac{x-f_{\min}}{f_{\max}-f_{\min}}$,  $a=m\left[\frac{m(1-m)}{v}-1\right]$, $b=(1-m)\left[\frac{m(1-m)}{v}-1\right]$,  $m=\frac{\lambda-f_{\min}}{f_{\max}-f_{\min}}$, $v=s\left[\frac{1}{(f_{\max}-f_{\min})}\right]^{2}$,  *f*_min_ = 1, *f*_max_ = *x*_max_ | λ > 0, *s* > 0 | 1 ≤ *x* ≤ *x*_max_ |
| Discretised stretched beta, 3 parameter form* | $f(z\vert\lambda,s,f_{\max})=\frac{\Gamma(a+b)}{\Gamma(a)\Gamma(b)}z^{(a-1)}(1-z)^{(b-1)}$ where:  $z=\frac{x-f_{\min}}{f_{\max}-f_{\min}}$,  $a=m\left[\frac{m(1-m)}{v}-1\right]$, $b=(1-m)\left[\frac{m(1-m)}{v}-1\right]$,  $m=\frac{\lambda-f_{\min}}{f_{\max}-f_{\min}}$, $v=s\left[\frac{1}{(f_{\max}-f_{\min})}\right]^{2}$,  *f*_min_ = 1 | λ > 0,  *s* > 0,  *f_max_*> 0 | 1 ≤ *x* ≤ *f*_max_ |
