# Supplementary material for: Does Litter Size Variation Affect Models of Terrestrial Carnivore Extinction Risk and Management?
Source: PLoS One. 2013 Feb 28;8(2):e58060. doi: 10.1371/journal.pone.0058060 (PMC3585178; doi:10.1371/journal.pone.0058060)
Supplement: Appendix S3 — Model descriptions for the three canid management scenarios used to illustrate the consequence of using different distributions to model litter size. (DOC) [file pone.0058060.s007.doc]

Appendix S3: Model descriptions for three canid management scenarios

*Model 1. A population viability analysis for the island fox on Santa Catalina Island, California.*

An annual, density-dependent, stochastic population viability analysis (PVA) of the Santa Catalina island fox, *Urocyon littoralis,* was written, based on [1] with initial parameter values taken from their model (Table 1). Mean litter size in their model was taken from [2]; we obtained the empirical litter size frequency data from [3]. Two subpopulations (east and west) were simulated over a 100-year period, with a catastrophe event occurring at a frequency of 20%, and a severity of an 80% reduction in survival. In this way, the model encapsulates a disease event (e.g. canine distemper virus). Breeding was density-dependent, and varied between both subpopulations. The proportion of females breeding at the carrying capacity for each subpopulation was determined according to equation (1) in [1]. Following [4], environmental variation was simulated by drawing age-specific mortality rates at the start of each year from a binomial distribution with a specified mean and standard deviation (Table 1) and demographic stochasticity in mortality was modelled with a binomial trial. The PVA [1] was run in VORTEX, and the same sequence of events was used [4] to create the model in R 2.14.0 (R Development Core Team 2011) to allow greater flexibility in specifying probability distributions. 10,000 replicates of the model were run for each of the 12 litter size probability distributions to establish 95% confidence intervals around a binomial outcome [5] of quasi-extinction probabilities for each metapopulation.

*Model 2. A stochastic simulation model to evaluate the efficacy of a control regime for rabies in urban foxes in Bristol.*

A monthly, stochastic, simulation model of the red fox, *Vulpes vulpes*, was constructed, based on [6], with initial parameter values taken from their model (Table 1). Litter size frequency data from Bristol [7] were used. Breeding was simulated in April (month 1 in this model), and one female per group was given an opportunity to breed. Age-specific mortality probabilities were drawn from a binomial distribution. Environmental variation was not included in this model. During month 7-12 juvenile males and females dispersed with set probabilities. The model was run for three years. Rabies was introduced by infecting all foxes within one group at the beginning of September in the first year, with a latency period of one month before becoming infectious. Neighbouring individuals were then infected with the following contact probabilities: within group infection 0.9, neighbouring cubs during summer 0.3, if male, to infect neighbouring females during winter 0.9, any other neighbour infection 0.6. The initial analysis determined that a control regime of 40% every two months, three months after disease introduction, for a total of 4 events would reduce the initial population size by 87%, therefore reducing the density to below the threshold for successful disease eradication. 10,000 replicates of the model were run for each of the 12 litter size probability distributions to establish 95% confidence intervals around a binomial outcome [5] of reaching this threshold.

*Model 3. A population viability analysis for the African wild dog*

An annual, stochastic PVA of the African wild dog, *Lycaon pictus*, was simulated, based on [8], with initial parameter values taken from their model (Table 1). Their model was run in VORTEX, and as in Model 1, the same sequence of events was used [4] to create the model in R 2.14.0 (R Development Core Team 2011). Litter size in their model was input as an empirical distribution based on data from [9], and we used these data to fit the 12 probability distributions used in our study. Following [4], environmental variation was simulated by drawing age-specific mortality rates at the start of each year from a binomial distribution with a specified mean and standard deviation (Table 1) and demographic stochasticity in mortality was modelled with a binomial trial. We simulated a small population of 20 individuals for 50 years, found from their PVA to be the most susceptible to extinction. Breeding was not density dependent, but at the start of each simulation it was assumed that the population was at carrying capacity [8], and following VORTEX [4], truncation was applied above this value by including a separate survival component. Two catastrophes were included, a mild and a severe, to simulate environmental events, or a disease outbreak respectively. An Allee effect was included by reducing each litter size draw by *k*(*P_t_* – *N*), where *P_t_* is the carrying capacity; and *k,* estimated to be 0.8 [10], is the slope of the relationship between pack size, *N*, and the number of pups recruited to yearling age. 10,000 replicates of the model were run for each of the 12 litter size probability distributions with and without an Allee effect to establish 95% confidence intervals around a binomial outcome [5] of quasi-extinction probabilities.

The results of all three replicated models were compared with the original model predictions to ensure accurate replication, except for Model 3 with the inclusion of an Allee effect, which the original model did not incorporate. All models were run in R 2.14.0 (R Development Core Team 2011), <http://www.R-project.org/>).

**Literature cited in Appendix S3**

1. Kohlmann SG, Schmidt GA, Garcelon DK (2005) A population viability analysis for the island fox on Santa Catalina Island, California. Ecol Model 183: 77-94.

2. Coonan T, Austin JG, Schwemm C (1998) Status and trend of island fox, San Miguel Island, Channel Islands National Park. Ventura, California: National Park Service Technical Report 98-01. pp. 1-27.

3. Coonan T, unpublished data.

4. Miller PS, Lacy RC (2005) VORTEX: a stochastic simulation of the extinction process. Version 9.50 user’s manual. Apple Valley, Minnesota: IUCN SSC Captive Breeding Specialist Group.

5. Hilborn, R, Mangel, M (1997) The ecological detective: confronting models with data. Princeton, Princeton University Press.

6. Smith GC, Harris S (1991) Rabies in urban foxes (*Vulpes vulpes*) in Britain: the use of a spatial stochastic simulation model to examine the pattern of spread and evaluate the efficacy of different control régimes. Philos Trans R Soc Lond B Biol Sci 334: 459-479.

7. Harris S unpublished data.

8. Ginsberg JR, Woodroffe R (1997) Extinction risks faced by remaining wild dog populations. In: The African wild dog: status survey and conservation action plan. Woodroffe R, Ginsberg J, Macdonald DW, eds. Gland: IUCN. pp 75-87.

9. Fuller TK, Kat PW, Bulger JB, Maddock AH, Ginsberg JR, Burrows R, McNutt JW, Mills MGL (1992) Population dynamics of African wild dogs. In: McCullough DR, Barrett RH, editors. Wildlife 2001: populations. London: Elsevier Science Publishers. pp. 1125-1139.

10. Vial F, Cleaveland S, Rasmussen G, Haydon DT (2006) Development of vaccination strategies for the management of rabies in African wild dogs. Biol Conserv 131: 180-192.

.
